# Normative Modelling of Brain Volume for Diagnostic and Prognostic Stratification in Multiple Sclerosis

**DOI:** 10.1101/2025.09.14.25335702

**Published:** 2025-11-27

**Authors:** Max Korbmacher, Ingrid Anne Lie, Kristin Wesnes, Eric Westman, Thomas Espeseth, Ole Andreas Andreassen, Lars T. Westlye, Stig Wergeland, Hanne Flinstad Harbo, Gro Owren Nygaard, Kjell-Morten Myhr, Einar August Høgestøl, Øivind Torkildsen

**Affiliations:** 1 Neuro-SysMed, Department of Neurology, Haukeland University Hospital, Norway; 2 Mohn Medical Imaging and Visualisation centre, Department of Radiology, Haukeland University Hospital, Norway; 3 Department for Radiography, Western Norway University of Applied Sciences, Norway; 4 Department of Neurology, Oslo University Hospital, Norway; 5 Department of Neurology, St. Olav’s Hospital, Trondheim University Hospital, Norway; 6 Department of Neurobiology, Care Sciences, and Society Karolinska Institutet, Sweden; 7 The Ageing Epidemiology Research Unit, School of Public Health, Imperial College London, London, UK.; 8 Department of Psychology, University of Oslo, Norway; 9 Faculty of Psychology, Oslo New University College, Norway; 10 Center for Precision Psychiatry, University of Oslo and Oslo University Hospital, Norway; 11 K.G. Jebsen-Centre for Neurodevelopmental disorders, University of Oslo, Norway; 12 Department of Clinical Medicine, University of Bergen, Norway; 13 Norwegian MS-Registry and Biobank, Helse Bergen, Haukeland University Hospital, Norway; 14 Institute for Clinical Medicine, University of Oslo, Norway

## Abstract

**Background.:**

Brain atrophy is a hallmark of multiple sclerosis (MS). For clinical translatability and individual-level predictions, brain atrophy needs to be put into context of the broader population, using reference or normative models.

**Methods.:**

Reference models of MRI-derived regional brain volumes were established from a large healthy control (HC) multi-cohort dataset (N=63 115, 51% females). The reference models were applied to N=362 people with MS with T_1_w-scans=953, with a follow-up time of up to 12 years (Median=3.72, MAD=3.75) to assess deviations from the reference, defined as Z-values. We assessed the overlap of deviation profiles and their stability over time using individual-level transitions towards or out of significant reference deviation states (|Z|>1.96). A negative binomial model was used for 1:1 propensity-matched case-control comparisons of the number of extreme deviations. Linear models were used to assess differences in Z-score deviations between MS and propensity-matched HCs, and associations with clinical scores at baseline and over time. The utilized normative BrainReference models, scripts and usage instructions are freely available.

**Findings.:**

We identified a temporally stable, brain morphometric phenotype of MS. The right and left thalamic volumes most consistently showed significantly lower-than-reference volumes in MS (25% and 26% overlap across the sample). The number of such extreme smaller-than-reference values was 2.70 in MS compared to HC (4.51 versus 1.67). Each extreme norm-deviation at baseline indicated stronger disability (Expanded Disability Status Scale: EDSS) longitudinally (standardized coefficient (β)=0.07, 95% CI 0.02 to 0.13, p=0.016), and at baseline (β=0.24, 95% CI 0.14 to 0.34, p<0.00001). At baseline, volumetric deviations in cortical and subcortical regions were associated with EDSS and FSS, but not PASAT (p> 0.05), with strongest effects in the hippocampi (β = −0.17 to −0.21, p≤ 0.036), thalamus (β = −0.18, p=0.044), putamen (β = −0.21 to −0.23, p≤0.004), and right amygdala (β = −0.17, p=0.036). Longitudinally, lower Z-values predicted EDSS via thalamic, hippocampal, and putamen volumes (β = −0.14 to −0.18, p≤0.043), whereas no significant predictors emerged for FSS or PASAT.

**Interpretation.:**

We present a heterogeneous brain phenotype of MS which is associated with clinical manifestations and particularly implicating the thalamus. The findings offer potential to aid diagnosis and prognosis of MS.

## Introduction

Reference values are an essential part of clinical practice across medical fields. Yet, imaging-based reference values are rarely applied in neurological practice, due to variability in results and a lack of technical standardisation and hence comparability. Recent advances in normative modelling allow to translate such models to neuroscientific and hence neurological contexts.^1,2^ Normative models serve to quantify the degree to which an individual measure deviates from the distribution of normative reference population.^1^ While previous studies have established normative models for common metrics of cortical thickness and surface area^1,3,4^ as well as subcortical volumes,^5^ models covering both cortical and subcortical volumes still needs to be provided. Here, we develop such models and apply them to a pooled multiple sclerosis (MS) cohort to characterize the brain morphometric phenotype of MS, which assumes that there are common brain characteristics across people with MS. Over the past decades, magnetic resonance imaging (MRI) research in MS has progressed from a primary focus on lesion detection to the recognition of brain atrophy as a central marker of disease progression. Earlier work demonstrated that atrophy occurs across MS phenotypes and exceeds the rates observed in normal ageing,^6–8^ though technical limitations restricted its application for short-term individual monitoring.^9^ More recent work provided additional insights into the interplay between MS-related neurodegeneration and ageing, where deep grey matter, and particularly thalamic atrophy was related to disability accumulation.^10^ Cortical atrophy, on the other hand, has been associated with cognitive decline.^7^ Alongside these advances, the broader clinical role of MRI has expanded beyond diagnosis to monitoring and treatment guidance, reflecting a shift in the field from viewing MRI primarily as a diagnostic tool to using it as a window into the long-term mechanisms of neurodegeneration and ageing in MS.^11^ The next step is to establish generalisable reference values, which are practically and clinically usable.

Hence, the application of normative brain models to MS has two important functions. First, the procedure can aid to test image-derived markers suggested by the literature, such as assumptions about cortical and thalamic atrophy,^6–8^ as well as to explore new potential imaging markers. Secondly, normative models can indicate individual image-based norm-deviations which are not visible by standard neuroradiological assessments, making them a potentially useful clinical tool. By overlapping individual deviation profiles, one is able to appropriately define heterogeneity across people with MS and to explore the potential of grey matter structure as an early prognostic and diagnostic MS biomarker.^12^ We assessed age and sex dependent norm-deviations from regional grey matter volumes across people with MS to establish a morphometric grey matter phenotype of MS. We assessed case-control differences of norm deviations and related the deviations to relevant clinical outcomes cross-sectionally and longitudinally.

## Methods

### Participants

We used seven international databases to assemble a large cross-sectional healthy control (HC) cohort (N=62 795 after removal of missing values; for information see Supplemental Table 1), of which N=351 were used to one-on-one age-, sex-, and intracranial volume (ICV)-match a longitudinal MS dataset (N=351 with available MRI data; 82.8% females; age range 18.5–67.6 years; T1w scans=953; median follow-up time=3.72 years, MAD=3.75) using propensity scores. The remaining N=62 414 HC (50.8% females, age range 6.0–90.1) were used for model training.

The MS cohort was assembled from two independent datasets collected across Norway. The first MS sample included 88 people with MS who participated in the omega-3 fatty acid in MS (OFAMS) multicentre clinical trial conducted between 2004–2008^13^. The trial entailed monthly MRI-acquisition, and clinical examination performed every 6 months over a 2-year period, followed by a single follow-up visit about 10 years after the original trial concluded. The attrition rate was low with 96.6% (85 of 88) completing the 10-year follow-up. The second MS dataset was collected at the Oslo university hospital (OUH) during clinical and study assessments since 2012 with standard follow-ups (N=302, T_1_w-scans=690), with recruitment at the first assessment.^14^ Both MS cohorts were clinically assessed for motor and cognitive impairments by evaluating a) disability, using the Expanded Disability Status Scale (EDSS),^15^ b) cognitive function, using Z-scores of the 3 second Paced Auditory Serial Addition Test (PASAT),^16^ and c) the level of fatigue, using the mean Fatigue Severity Scale (FSS) score.^17^ EDSS scores were available at all MRI timepoints, however, there was systematic missingness of PASAT and FSS scores in parts of the cohort, as these assessments were not used at all recordings. All data collections and usage were approved by respective ethical review boards, and informed consent forms were obtained (see Supplemental Note 1).

### Magnetic resonance imaging (MRI) and data processing

T_1_-weighted MRI data were obtained using various scanners, sites, and field strengths (1.5T or 3T). Acquisition protocols and machines were stable over time, however, due to the long-follow up time of the multi-centre study (OFAMS), scanner software was updated during the study period. An overview of the acquisition protocols and MRI sequence information can be retrieved from the original studies (HC: Supplemental Table 1, MS OFAMS data: Supplemental Table 2, original studies^13,14^).

In the OFAMS multi-centre study, the acquisition protocol included the following MRI sequences: a T2-weighted 3D sagittal fluid attenuated inversion recovery (FLAIR) (resolution: 1 × 1 × 1 mm3, echo time (TE)/repetition time (TR)/inversion time (TI) = 386/5000/1.65–2.2 ms) and a postcontrast T1-weighted 3D sagittal magnetization-prepared rapid gradient echo sequence (resolution: 1 × 1 × 1mm3, TE/TR/TI = 2.28/1800/900 ms, flip angle 8°). See Supplemental Table 2 for site-specifc information on the OFAMS study. While MRI systems varied for OFAMS data, all data were collected on a single scanner in Oslo using a 3D sagittal brain volume (BRAVO) sequence for pre- and post-gadolinium contrast agent administration (1 × 1 × 1 mm resolution, TR = 8.16 ms, TE = 3.18 ms, TI = 450 ms, flip angle (FA) = 12°), and a 3D FLAIR sequence (1 × 1 × 1.2 mm resolution, TR = 8000 ms, TE = 127.25 ms, TI = 2240 ms) using a Discovery MR750 MRI (GE Medical Systems). After alignment to Montreal Neurological Institute (MNI) 152 standard space, white and grey matter segmentation, N4-bias field correction and linear, rigid body registration of T_2_-weighted (FLAIR) images to T_1_-weighted images using FSL FLIRT, T_1_-weighted lesions were manually segmented using ITK-SNAP. T_2_-weighted lesions were then manually segmented by two MRI technicians (with more than years of experience in MS lesion segmentation), with subsequent segmentation quality control by an experienced neuroradiologist. The resulting lesion masks were subsequently overlayed onto the co-registered T_1_-weighted and FLAIR images for editing, to include any T_2_-hyperintense lesion changes (i.e., new lesions, enlarging lesions, or decreasing lesions) in the follow-up scans. Any discrepancies in co-registrations that were visible between sessions were corrected manually using the ITK-SNAP automated registration tool before follow-up lesion mask edits. Lesion counts and volumes were extracted from lesion masks using fslmaths (https://fsl.fmrib.ox.ac.uk/fsl/fslwiki/Fslutils). T_2_-hyperintense lesion masks were used to fill longitudinally co-registered T_1_-weighted images with the lesion_filling function implemented FSL (version 5.0.10) to improve volume measurements by reducing intensity contrast within known lesion areas. An upper intensity threshold at the 98th percentile from gadolinium-enhanced regions was used to identify lesions to be filled. After lesion-filling, regional brain volumes were extracted using the longitudinal pipeline of FreeSurfer for the longitudinal data (v7.1.1 OFAMS and v6.0.0 for Oslo data) including people with MS, and the cross-sectional FreeSurfer pipeline for cross-sectional HC data (multiple versions, see Supplemental Table 1), and then averaged across the brain regions specified in the Desikan-Killiany atlas.^18^ For training data harmonisation, we applied Combat, which was originally developed for batch effects in laboratory samples^19^ and recently extended to neuroimaging data.^20^ To not introduce incorrect group differences through harmonizing the test data,^21^ particular in the context of the small number of subjects per scanner site,^22^ we did not harmonise the test data. However, as a quality control measure, we report results from harmonised test data in the Supplement (Supplemental Figures 3–6 and 12–13, Supplemental Note 4).

### Statistical analysis

**Figure 1.**
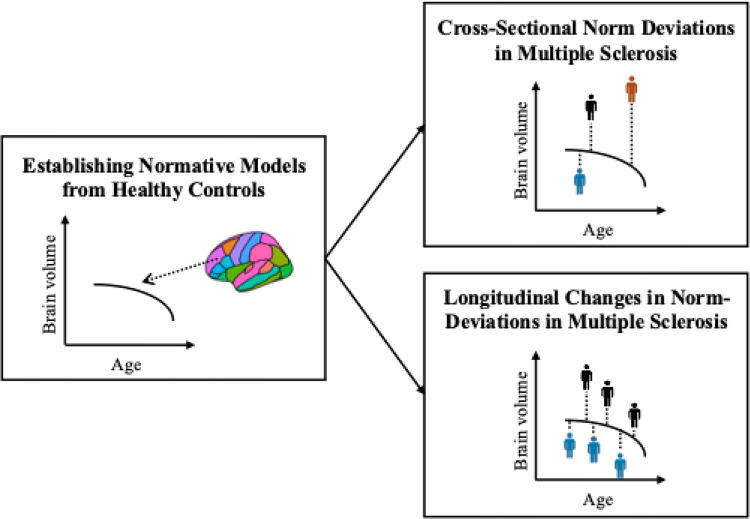
Analysis workflow. First, normative models were established on a large cohort of healthy controls covering the lifespan (6.0–90.1 years of age). Second, the reference models were applied to the MS cohort to assess deviations from the norm cross-sectionally (upper right panel) and over time (lower right panel). The colour of the mannequins in the right panels represent a single individual with multiple sclerosis.

An overview of the full analysis workflow is presented in Figure 1.

Sex-stratified cortical and subcortical regional brain volumes were extracted using the FreeSurfer implementation of the Desikan-Killiany atlas and subcortical parcellation schemes. For model training, we used multivariate fractional polynomial regression,^23^ trained on regional brain volumes of a large HC cohort, comprised of seven cohorts (N=62 467, after removal of missing values). Models were trained for each sex separately, and trained for each brain region (i), where regional brain volumes were predicted from a linear effect of ICV, to account for confounding effects of head size, a fractional polynomial (p) of age (with maximum degree m=2), and an error term (u), with b_0-n_ indicating the regression coefficients.


$${\text{region}}_{i}=b_{0}+b_{1}ICV_{i}+b_{2}{\text{age}}^{p}+u_{i}$$

Model performance across regions was similar in training data R^2^ = 0.44±0.16, r = 0.66±0.13, RMSE (%) = 13.6±3.29 and MAE (%) = 10.6±2.58 compared to propensity matched HCs R^2^ = 0.34±0.19, r = 0.63±0.12, RMSE (%) = 14.80±3.96 and MAE (%) = 11.60±3.18. For an overview of region-wise model performance metrics see Supplemental Figure 7 and 8. The models were then used to predict the brain volumes per region in people with MS (N=362, T_1_w-scans=953), as well as in propensity-score matched cross-sectional HC sample (based on ICV, sex, and age, N=351). Z-scores, representing norm deviations, were calculated from the true volumes, predicted volumes, and model error (RMSE). First, we quantified the total number of regional significant deviations or extreme lower-than-reference values, defined by Z < −1.96. The number of extreme lower-than-reference values (XLTRV) per individual was then compared between people with MS and HC, using negative binominal regression to account for overdispersion. XLTRV was then associated with EDSS, PASAT and FSS scores at baseline, using cross-sectional linear models, and longitudinally, using linear random intercept models, controlling for disease duration (DD):

$$\text{Clinical}=b_{0}+b_{1}\text{XLTRV}+DD+u$$

Note that, by training subgroup specific models and including ICV in model-training, the Z-scores in test data are already sex, age, and ICV-adjusted; however, we corrected for DD. Moreover, we did not include information on treatment in the modelling as the treatment plans were not comparable to each other and highly variable (Supplemental Tables 4–5). This was due to the historic developments of treatments and treatment strategies. While there were 119 relapses, this number was restricted to 43 participants during the entire study period. Additionally, a large portion of these relapses were present during the first years of the OFAMS clinical trial (65 relapses across 37 patients) making this variable unsuitable as a covariate in longitudinal analyses. However, for quality control, we assessed the relationship between both Z-values and the number of extreme deviations with the number of relapses at baseline, presenting no significant relationships (Supplemental Note 3).

There was a small increase of T_1_-weighted lesions over the first two years of the study period, with this period being chosen for comparability between samples. There were only 2 new T_1_-weighted lesions in Oslo data and 18 in OFAMS data. The count of T_2_-weighted lesions fluctuated over time. We did not observe associations between T_2_-weighted lesions and Z-values or extreme deviations, however significant associations were found for T_1_-weighted lesions (Supplemental Note 3). Note however that T_1_-weighted lesions were not a significant predictor of EDSS and did not improve model performance compared to the number of extreme deviations (Supplemental Note 3). We did hence not include this variable in further analyses.

Using the same logic from above, we assessed the impact of age, EDSS, PASAT and FSS on normative deviations (Z) cross-sectionally and longitudinally.


$$\text{Clinical}=b_{0}+b_{1}Z_{\text{region}}+DD+u$$

Since extreme deviations were defined as |Z|>1.96, we defined stability of such deviations as the absence of any time transitions inside or outside the extreme deviation state (whether Z>1.96 or Z<−1.96). For comparability, we report standardized regression coefficients or effect sizes. The significance-level was set at a conventional alpha level=0.05, and the Benjamini-Hochberg (FDR) correction for multiple comparisons^24^ was applied for all tests. MRI data were used when also data on sex and age were available. This allowed sex-specific predictions and calculation of Z-scores using the normative models. Missingness at random was addressed using multiple imputation, where appropriate. We used multivariate imputation by chained equations, using a single iteration of predictive mean matching. Due to missingness, least data were available for cross-sectional correlations between FSS scores and brain volumes. Here, the minimal observable effect was f^2^=0.25 (corresponding to R^2^=0.20, and Pearson’s r=0.45), at 80% power, alpha=0.05, 3 nominator and 43 denominator degrees of freedom. For statistical analyses, R version 4.5.0 was used. To represent spatial statistics for the examined brain areas, we used the ggseg R package.^25^

## Results

### Descriptives

After removing missing data on MRI, sex, and age, 72 (7.76%) EDSS scores across all sessions, which were missing at random, were imputed, resulting in data from 953 MRI sessions from 362 people with MS. At baseline, the 351 people with MS with available MRI data and matched with HC were aged 38.6 (9.7) years, with 250 (71.2%) being females, and an average disease duration of 4.66 (6.15) years, average EDSS of 2.0 (1.2), PASAT 46.8 (9.2) and FSS of 4.72 (1.48). There was a total of 119 relapses across 43 participants across the entire study period. Additional sub-sample baseline demographics can be found in Supplemental Table 3, and the distribution of Z-scores in Supplemental Figure 11, and extreme Z-score deviations in Supplemental Figure 1.

### Number of extreme lower-than-reference deviations from normative brain models

**Figure 2.**
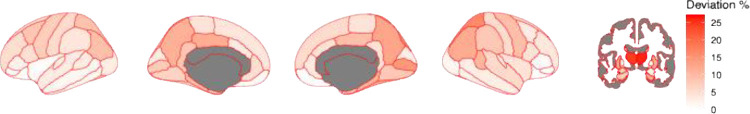
Overlap of significant deviations indicating lower brain volumes (Z<−1.96) across people with multiple sclerosis at baseline. Darker colours indicate a higher percentage of people with MS overlapping in deviations in the respective brain region.

At baseline, the largest overlap of extreme lower-than-reference volumetric deviations across people with MS could be shown bilaterally in the thalami (25% and 26%, see Supplemental Figure 1 for distributions), followed by the right superior parietal area (16%) and pericalcarine (14%; Figure 2). Multiple other regional presented extreme deviations in more than 10% of the people with MS, including the praecuneus, fusiform area, putamen, inferior parietal area, posterior cingulate, putamen, hippocampus and parahippocampal area. These regions also presented the strongest deviations, measured by Z-scores (Supplemental Figure 1). In contrast, brain volumes among HC corresponded to reference levels (Supplemental Figure 2). Examples for individual-level profiles for Z-scores can be found in Supplemental Figure 9 and for Z-score-based extreme deviations in Supplemental Figure 10.

Note that these deviations remained stable over time, indicated by maintaining the state of extreme or non-extreme deviations over time and across regions. Most people with MS (Mean 94.81% (SD 3.16%) presented no change in the total number of extreme deviations, 2.96% (SD 1.47%) of the sample presented worsening compared to the norm: the transition towards lower-than-reference brain volumes, and 2.39% (SD 1.67%) presented improvements, indicated by transitions from extreme lower-than-reference values towards non-extreme or extremely positive deviations. See Supplementary Figure 9 for individual-level example brain maps. Note that the number of HC who had an accumulation of extreme lower-than-reference values (2.39% (SD 1.67%)) was even lower than in people with MS (Supplemental Note 2).

At baseline, extreme lower-than-reference brain volumes indicated higher EDSS (β=0.24, 95% CI 0.14 to 0.34, p<0.00001), but not PASAT (p=0.155), FSS (p=0.131), or disease duration (p=0.727). Similarly, longitudinally, the number of deviations increased with EDSS (β=0.07, 95% CI 0.02 to 0.13, p = 0.016), but was not associated with PASAT (p=0.591), and FSS (p=0.255). Group-level cross-sectional case-control comparisons presented that people with MS had nearly three times the number of norm-deviations (4.51, SE 4.95) compared to HC (1.67, SE 2.67), indicated by an incidence rate ratio of 2.70, 95% CI 2.21 to 3.30, p < 0.00001, and lower Z-scores than HC (average of regional mean absolute ΔZ=−0.22 (SE 0.48), d=−0.48, 95% CI −0.17 to 0.79, p=0.003). The regions with the largest negative Z-scores across people with MS, indicating smaller brain volumes, were the thalami (Z_left_=−1.16, Z_right_=−0.84), praecuneus (Z_left_=−0.94, Z_right_=−0.94), posterior cingulate (Z_left_=−0.91, Z_right_=−0.70), and putamen (Z_left_=−0.87, Z_right_=−0.16).

### Magnitude of norm-deviations and clinical associations

**Figure 3.**
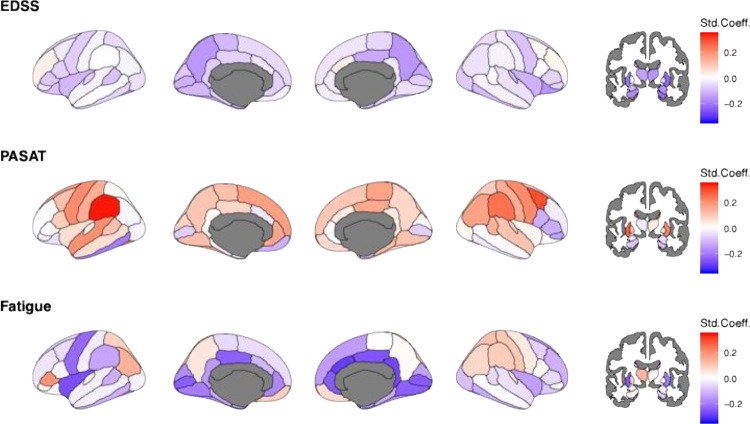
Baseline associations of age, EDSS, PASAT, and FSS on regional Z-scores. Std.Coeff.=standardized coefficient, EDSS= Expanded Disability Status Scale, PASAT= Paced Auditory Serial Addition Test, FSS=Fatigue Severity Scale. Note that only N=111 complete cases were available for PASAT and N = 119 for FSS scores. Considering the high level of missingness, imputation was not executed. Darker red colour indicates positive and darker blue colour negative deviations from the norm, representing larger and smaller brain volumes compared to the reference. White colour indicates effects equal zero. Grey colour indicates uncorrected p>0.05.

At baseline, multiple regional deviations were significantly associated with EDSS and FSS, but not PASAT (p_FDR_>0.05; Figure 3). Significant associations were found between EDSS and volumetric deviations in cortical and subcortical regions, including the hippocampi (left: β_EDSS_=–0.17 95% CI −0.27 to −0.07; p_FDR_=0.036; right: β_EDSS_=–0.21 95% CI −0.31 to −0.11; p_FDR_=0.004), right thalamus (right: β_EDSS_=–0.18 95% CI −0.29 to −0.07; p_FDR_=0.044), putamen (left: β_EDSS_=–0.21 95% CI −0.31 to −0.11, p_FDR_=0.004, right: β_EDSS_=–0.23 95% CI – 0.33 to −0.07, p_FDR_=0.002), and right amygdala (β_EDSS_=–0.17 95% CI −0.28 to −0.07, p_FDR_=0.036).

Deviations from the cortical volumes in the left supramarginal gyrus (β_EDSS_=0.31 95% CI 0.13 to 0.48, p_FDR_=0.010) predicted PASAT, and in the right isthmus cingulate (β_EDSS_=–0.33 95% CI −0.50 to −0.16, p_FDR_=0.010) predicted FSS.

**Figure 4.**
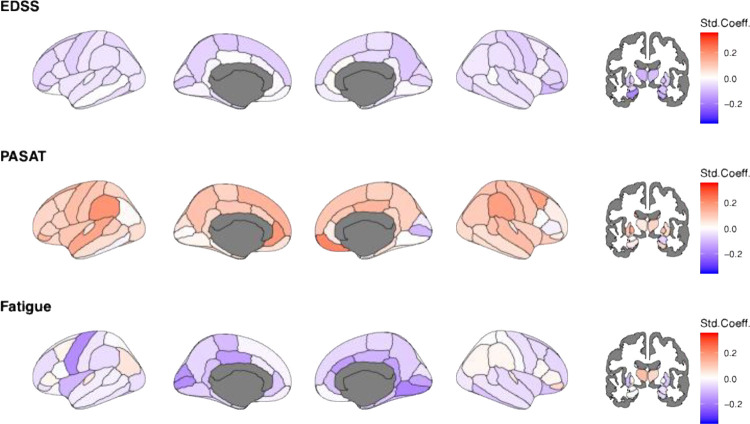
Longitudinal association of age, EDSS, PASAT, and FSS with regional Z-scores Std.Coeff.=standardized coefficient, EDSS= Expanded Disability Status Scale, PASAT= Paced Auditory Serial Addition Test, FSS=Fatigue Severity Scale. Age and EDSS were available for all participants, for N=107 (222 sessions) for PASAT and N=110 (214) for FSS, with this high level of missingness not allowing for imputation. Darker red colour indicates positive and darker blue colour negative deviations from the norm, representing larger and smaller brain volumes compared to the reference. White colour indicates effects equal zero. Grey colour present regions which were not assessed. All associations were significant *before* FDR-corrections. Corrected, significant relationships are reported in-text.

In longitudinal analyses, lower Z-values, indicating lower-than-reference brain volumes, affected widespread and yet function-specific areas, with significant relationships detected for EDSS (Figure 4). EDSS could be predicted by volumes in the thalami (left: β_EDSS_=–0.15 95% CI −0.23 to −0.08; p_FDR_=0.006; right: β_EDSS_=–0.14 95% CI −0.22 to −0.07; p_FDR_=0.021), hippocampi (left: β_EDSS_=–0.16 95% CI −0.22 to −0.10; p_FDR_<0.001; right: β_EDSS_=−0.15 95% CI −0.22 to −0.07; p_FDR_=0.021), and the left putamen (β_EDSS_=−0.18 95% CI −0.26 to −0.11; p_FDR_=0.043). No reference values could significantly predict FSS or PASAT scores.

The results remained unchanged when applying a harmonisation strategy directly to the test data, except for counter-intuitive findings of lower levels of PASAT being associated with negative Z-scores (brain volumes smaller than the norm; see Supplemental Figures 3–6).

## Discussion

By applying normative models, trained on over 62 000 healthy individuals, to an MS cohort with several longitudinal follow-ups, we presented that extreme brain volume deviations are heterogeneously distributed across the brain. The thalamus was the region with the largest overlap of extreme deviation across patients. Around one fourth of the examined MS cohort presented extremely lower-than-reference thalamic volumes, suggesting the thalamus as the most representative region characterising MS. Note however, that the pattern of deviations from reference values was also highly heterogeneous. This regional pattern of reduced brain volume compared to the healthy population norm associated with disability, cognitive dysfunction and fatigue, which are all key symptoms of MS.

These findings align with previous literature implicating thalamic and deep grey matter atrophy in MS pathophysiology, progression and the same clinical outcomes as presented here.^6–8^ However, by expressing these deviations as age- and sex-adjusted Z-scores relative to normative reference distributions, we provide interpretable, individual-level metrics.^1^ On the group level, the extent of regional norm-deviations, indicating lower brain volumes, rather than the number of affected brain areas, more consistently explained variance in clinical measures. Subcortical deviations, particularly in the thalami and the right putamen, were significantly associated with physical disability, while frontal cortical regions were associated with cognitive function and fatigue. While correlations between thalamic characteristics and clinical assessments of disability are known, the frontal cortex associations with cognitive function and fatigue are sparsely reported. However, the presented results reinforce the neuroanatomical specificity of MS symptomatology.^6–8^ That way, norm-deviations can be used to stratify patients based on an expectable phenotype, where conventional radiological markers do not appear diagnostic or prognostically useful. Moreover, norm-deviations, either quantified as Z values or total counts of extreme deviations provide stronger associations with clinical outcomes and a clearer morphometric profile than simple volumetric assessments.^26^

Our findings extend previous work on grey matter atrophy in MS by placing individual-level morphometric variation in a normative context. Compared to age- and sex-matched HC, people with MS had nearly three times the number of significant deviations in brain volumes, and these deviations persisted over time. The stability of deviations per brain region across time and individuals, especially in subcortical structures, suggests a general MS-specific brain morphometric phenotype. This is the first time such phenotype has been established, which might bear potential for early diagnosis, disease monitoring and future clinical trial outcomes. Deviations from reference values can serve to assess the risk of the development of MS from radiologically isolated syndrome, from MRI examinations in general, or be a marker for progression independent of relapse activity.

Furthermore, longitudinal associations showed that the time since baseline was linked to growing divergence from the norm, suggesting that normative models could also help disentangle MS- and age-related atrophy. A proposed disease-specific process might be that the thalamus, as a relay station of major white matter tracts, loses volume as axons degenerate. Frontal degeneration might indicate another biological process, for example, accelerated atrophy compared to non-pathological agers due to increased neuroinflammation. Notably, we observed minimal changes in deviation status (from significant to non-significant or vice versa) over time, reinforcing the temporal robustness of these markers. Still, in every tenth case, transitions in the deviation status were recorded in the thalami, highlighting the importance of repeated assessments in tracking disease progression. Normative modelling offers several advantages over conventional group-level analyses. It provides information for single individuals, allowing identification of subclinical deviations invisible to visual inspection or group averaging.^27^ This is particularly relevant in MS, where early and accurate detection of grey matter atrophy could facilitate timely therapeutic adjustments.^27,28^ Moreover, the integration of normative deviations into clinical workflows could complement conventional measures such as lesion load and global brain volume metrics. By enabling a regionally resolved and person-specific assessment of neurodegeneration, normative modelling could support future precision medicine approaches in MS.

These findings also open avenues to better understand cases with diagnostic challenges, and for understanding the heterogeneity of disease trajectories. Similar to previously reported deviation patterns in psychiatric disorders,^29,30^ we found extensive heterogeneity across people with MS, yet with larger region-specific overlaps than in psychiatric disorders, especially in the thalamus. This underscores the potential of brain imaging assessments in MS and their further epidemiological investigation.

This study has several strengths, including the use of large, multi-site normative data, harmonised processing pipelines, robustness checks and independent longitudinal MS samples. Nonetheless, some limitations must be noted. First, the test data presented in the main manuscript used for normative comparisons were not harmonised, which may introduce site-specific noise. On the other hand, this choice increases ecological validity and clinical applicability, and the results were stable also when computationally harmonising the data prior to analyses. Second, clinical data (particularly PASAT and FSS) were sparsely available and missing at random, limiting the statistical power for some analyses and precluding imputation. Future studies with complete longitudinal assessments of cognitive and fatigue are needed to validate and extend these findings. Additionally, while we used validated segmentation pipelines, volume estimates may be affected by scanner and MRI protocol variability, particularly in longitudinal settings. Recent evidence suggests that lesion filling is superior to non-filling when estimating regional brain volumes and brain age, a type of normative modelling approach where age is the prediction target.^31^ However, since we found an association between T_1_-weighted lesions and both Z-values and the number of extreme deviations, we cannot differentiate whether the presence of lesions biases volumetric estimates in themselves, or whether there is a biologically meaningful associations. However, extreme deviations were stronger and significantly associated with EDSS, which was not the case for T_1_-weighted lesions, there was no significant interaction effect between EDSS and T_1_-weighted lesions, while multicollinearity was low (Supplemental Note 3). Still, our sensitivity analyses using harmonised data produced concordant trends, reinforcing the robustness of the primary findings (see Supplemental Note 4 for deviations). Future studies might consider additional segmentations of thalamic subfields or the medulla to further explore MS disability associated regions with population reference values.

Our findings indicate that normative modelling could be a valuable tool for understanding MS. By measuring how an individual’s brain structure deviates from a healthy reference, we observed a consistent pattern linked to clinical symptoms. On the group level, deviations in the thalamus and frontal lobe appear to be significant imaging markers associated with disability, fatigue, and processing speed. While these volumetric deviations correlate with clinical status, more research is needed to confirm their value for diagnosis or prognosis. Nevertheless, these results suggest that integrating normative modelling into MS neuroimaging pipelines could support the early detection and monitoring of neurodegeneration.

## Supplementary Material

Supplement 1

## Data Availability

Summary statistics and utilized code can be found at https://osf.io/d6nfq. Trained models can be found at https://doi.org/10.17605/OSF.IO/6R8DY. Multiple of the utilized dataset are sensitive, require IRB approval for usage, and can therefore not be openly shared.
