# Normative Modelling of Brain Volume for Diagnostic and Prognostic Stratification in Multiple Sclerosis

**DOI:** 10.1101/2025.09.14.25335702

**Published:** 2026-02-03

**Authors:** Max Korbmacher, Ingrid Anne Lie, Kristin Wesnes, Eric Westman, Thomas Espeseth, Ole Andreas Andreassen, Lars T. Westlye, Stig Wergeland, Hanne Flinstad Harbo, Gro Owren Nygaard, Kjell-Morten Myhr, Einar August Høgestøl, Øivind Torkildsen

**Affiliations:** 1 Neuro-SysMed, Department of Neurology, Haukeland University Hospital, Norway; 2 Mohn Medical Imaging and Visualisation centre, Department of Radiology, Haukeland University Hospital, Norway; 3 Department for Radiography, Western Norway University of Applied Sciences, Norway; 4 Department of Neurology, Oslo University Hospital, Norway; 5 Department of Neurology, St. Olav’s Hospital, Trondheim University Hospital, Norway; 6 Department of Neurobiology, Care Sciences, and Society Karolinska Institutet, Sweden; 7 The Ageing Epidemiology Research Unit, School of Public Health, Imperial College London, London, UK; 8 Department of Psychology, University of Oslo, Norway; 9 Faculty of Psychology, Oslo New University College, Norway; 10 Center for Precision Psychiatry, University of Oslo and Oslo University Hospital, Norway; 11 K.G. Jebsen-Centre for Neurodevelopmental disorders, University of Oslo, Norway; 12 Department of Clinical Medicine, University of Bergen, Norway; 13 Norwegian MS-Registry and Biobank, Helse Bergen, Haukeland University Hospital, Norway; 14 Institute for Clinical Medicine, University of Oslo, Norway

## Abstract

Interpreting brain structure at the individual level remains a major challenge in neuroimaging, as population variability across age and sex limits the clinical utility of group-level findings. Here, we develop large-scale normative models of regional cortical and subcortical brain volumes from more than 62,000 healthy individuals across the lifespan and apply them to multiple sclerosis (MS) to enable individualised, reference-based assessments of grey matter morphology. We identify a temporally stable yet heterogeneous morphometric phenotype of MS, expressed as concordant deviations from age- and sex-adjusted reference values. Individual deviation profiles are clinically informative: both the magnitude and cumulative burden of lower-than-reference volumes are associated with disability cross-sectionally and longitudinally. Moreover, the profiles can be translated into interpretable stratification rules linked to disability trajectories and relapse dynamics. This work reframes known structural abnormalities into stable, individual-level deviation profiles, demonstrating how normative modelling can move neuroimaging beyond group averages toward clinically interpretable inference. Together, these findings establish a generalisable framework for translating population-level neuroimaging data into individual-level phenotypes with potential beyond multiple sclerosis.

## 1. Introduction

Interpreting biological measurements at the individual level remains a fundamental challenge across medicine and neuroscience, where population-level variability often obscures clinically meaningful deviations. Yet, imaging-based reference values are rarely applied in neurological practice, as technical and biological variability limit comparability across studies. Recent advances in normative modelling have begun to address these challenges, enabling the translation of population-level neuroimaging data into neuroscientific and clinical contexts.^1,2^ Normative models quantify the degree to which an individual measure deviates from the distribution of normative reference populations.^1^ Previous studies have established normative models for common metrics of cortical thickness and surface area^1,3,4^ as well as subcortical volumes,^5^ yet comprehensive models that jointly characterise both cortical and subcortical regional volumes with direct clinical applicability are still limited. Addressing this gap would move the field beyond group averages toward individualised phenotyping that can support diagnosis, prognosis, and monitoring. Multiple sclerosis (MS) offers a well-characterised disease context in which to evaluate whether normative modelling can reveal concordant patterns of grey matter deviation.

Over the past decades, magnetic resonance imaging (MRI) research in MS has evolved from a primary focus on lesion detection to the recognition of brain atrophy as a central marker of disease progression. Atrophy occurs across MS phenotypes and exceeds the rates observed in normal ageing,^6–8^ though technical limitations restricted its application for short-term individual monitoring.^9^ While deep grey matter, and particularly thalamic atrophy, presented associations with disability accumulation,^10^ cortical atrophy has been associated with cognitive decline.^7^ Alongside these advances, the broader clinical role of MRI has expanded beyond diagnosis to monitoring and treatment guidance, reflecting a shift in the field from viewing MRI primarily as a diagnostic tool to using it as a window into the long-term mechanisms of neurodegeneration and ageing in MS.^11^ However, these findings are largely based on group-level analyses and therefore provide limited guidance for interpreting individual patient scans, representing a major barrier to clinical translation. The next step is to establish generalisable, practically usable reference distributions against which individual scans can be contextualised.

Normative modelling directly addresses this clinical interpretability gap by estimating individual deviation profiles relative to age- and sex-adjusted reference distributions, thereby enabling a) rigorous testing of imaging markers proposed by the literature (e.g., cortical and thalamic atrophy),^6–8^ and b) discovery of additional, potentially overlooked markers that may not be apparent to standard neuroradiological assessment. By aggregating single-subject deviation profiles, one can quantify heterogeneity across people with MS (pwMS), delineate a reproducible morphometric phenotype characterised by coordinated deviations across distributed grey matter systems, and assess its clinical relevance for disability and progression.^12^ Such a framework is disease-agnostic and scalable: it aims to translate population-level neuroimaging into individualised brain phenotypes that are interpretable in clinical settings.

Here, we develop large-scale normative models for regional cortical and subcortical grey matter volumes derived from MRI and apply them to a pooled MS cohort to establish a morphometric phenotype of MS at the individual level. Using a reference dataset of N = 63,115 healthy controls (51% female) to derive age- and sex-adjusted expectations, we assess norm-deviations in N = 362 pwMS across 953 T1-weighted MRI scans spanning up to 12±3.72 years. We address three goals: i) to determine whether MS expresses a morphometric phenotype reflected by concordant deviations across deep and cortical grey matter; ii) to characterise heterogeneity in deviation profiles and quantify the burden of lower-than-reference regional volumes at the person level; and iii) to establish the clinical relevance of these deviations for disability and disease course, cross-sectionally and longitudinally. Together, this work provides a generalisable framework for individualised, reference-based interpretation of brain structure that is directly extensible to other neurological and psychiatric conditions.

## 2. Methods

### 2.1 Participants

#### 2.1.1 Healthy control cohort

We used seven international databases to assemble a large cross-sectional healthy control (HC) cohort (originally: N=63,115, N=62,795 after removal of missing values; for information see Supplemental Table 1), of which N=351 were used to one-on-one age-, sex-, and intracranial volume (ICV)-match a longitudinal MS dataset (N=351 with available MRI data; 82.8% females; age range 18.5–67.6 years; T1w scans=953; median follow-up time=3.72 years, MAD=3.75) using propensity scores at baseline. The remaining N=62,444 HC (50.8% females, age range 6.0–90.1) were used for model training.

#### 2.1.2 Multiple sclerosis cohort

The MS cohort was assembled from two independent datasets collected across Norway diagnosed using the McDonald criteria.^13^ The first MS sample included 88 pwMS who participated in the omega-3 fatty acid in MS (OFAMS) multicentre clinical trial conducted between 2004–2008^14^. The trial entailed monthly MRI-acquisition, and clinical examination performed every 6 months over a 2-year period, followed by a single follow-up visit about 10 years after the original trial concluded. The attrition rate was low with 96.6% (85 of 88) completing the 10-year follow-up. The second MS dataset was collected at the Oslo university hospital (OUH) during clinical and study assessments since 2012 with standard follow-ups (N=302, T_1_w-scans=690), with recruitment at the first assessment.^15^ Both MS cohorts were clinically assessed for motor and cognitive impairments by evaluating a) disability, using the Expanded Disability Status Scale (EDSS),^16^ b) cognitive function, using Z-scores of the 3 second Paced Auditory Serial Addition Test (PASAT),^17^ and c) the level of fatigue, using the mean Fatigue Severity Scale (FSS) score.^18^ EDSS scores were available at all MRI timepoints, however, there was systematic missingness of PASAT and FSS scores in parts of the cohort, as these assessments were not used at all recordings. All data collections and usage were approved by respective ethical review boards, and informed consent forms were obtained (see Supplemental Note 1).

### 2.2 Magnetic resonance imaging (MRI)

#### 2.2.1 MRI acquisition

T_1_-weighted MRI data were obtained using various scanners, sites, and field strengths (1.5T or 3T). Acquisition protocols and machines were stable over time, however, due to the longfollow up time of the multi-centre study (OFAMS), scanner software was updated during the study period. An overview of the acquisition protocols and MRI sequence information can be retrieved from the original studies (HC: Supplemental Table 1, MS OFAMS data: Supplemental Table 2, original studies^14,15^).

In the OFAMS multi-centre study, the acquisition protocol included the following MRI sequences: a T2-weighted 3D sagittal fluid attenuated inversion recovery (FLAIR) (resolution: 1×1×1 mm3, echo time (TE)/repetition time (TR)/inversion time (TI) = 386/5000/1.65–2.2 ms) and a postcontrast T_1_-weighted 3D sagittal magnetization-prepared rapid gradient echo sequence (resolution: 1 × 1 × 1mm3, TE/TR/TI = 2.28/1800/900 ms, flip angle 8°). See Supplemental Table 2 for site-specific information on the OFAMS study.

While MRI systems varied for OFAMS data, all data were collected on a single scanner in Oslo using a 3D sagittal brain volume (BRAVO) sequence for pre- and post-gadolinium contrast agent administration (1×1×1 mm resolution, TR = 8.16 ms, TE = 3.18 ms, TI = 450 ms, flip angle (FA) = 12°), and a 3D FLAIR sequence (1×1×1.2 mm resolution, TR = 8000 ms, TE = 127.25 ms, TI = 2240 ms) using a Discovery MR750 MRI (GE Medical Systems).

#### 2.2.2 MRI processing

After alignment to Montreal Neurological Institute (MNI) 152 standard space, white and grey matter segmentation, N4-bias field correction and linear, rigid body registration of T_2_-weighted (FLAIR) images to T_1_-weighted images using FSL FLIRT, T_2_-lesions were segmented using FSL’s (version 5.0.10) lesion segmentation tool. Lesion counts and volumes were extracted from lesion masks using fslmaths (https://fsl.fmrib.ox.ac.uk/fsl/fslwiki/Fslutils). T_2_-hyperintense lesion masks were used to fill longitudinally co-registered T_1_-weighted images with the lesion_filling function implemented in FSL (version 5.0.10) to improve volume measurements by reducing intensity contrast within known lesion areas. An upper intensity threshold at the 98th percentile from gadolinium-enhanced regions was used to identify lesions to be filled. After lesion-filling, regional brain volumes were extracted using the longitudinal pipeline of FreeSurfer for the longitudinal data (v7.1.1 OFAMS and v6.0.0 for Oslo data) including pwMS, and the cross-sectional FreeSurfer pipeline for cross-sectional HC data (multiple versions, see Supplemental Table 1), and then averaged across the brain regions specified in the Desikan-Killiany atlas.^19^ For training data harmonisation, we applied Combat, which was originally developed for batch effects in laboratory samples^20^ and recently extended to neuroimaging data.^21^ To not introduce incorrect group differences through harmonizing the test data,^22^ particular in the context of the small number of subjects per scanner site,^23^ we did not harmonise the test data. This approach preserves biological variance in the test data and avoids introducing artificial group differences when the number of subjects per site is small. As an additional quality control measure, we report results from harmonised test data in the Supplement (Supplemental Figures 3–6 and 12–13, Supplemental Note 4).

### 2.3 Statistical analysis

**Figure 1.**
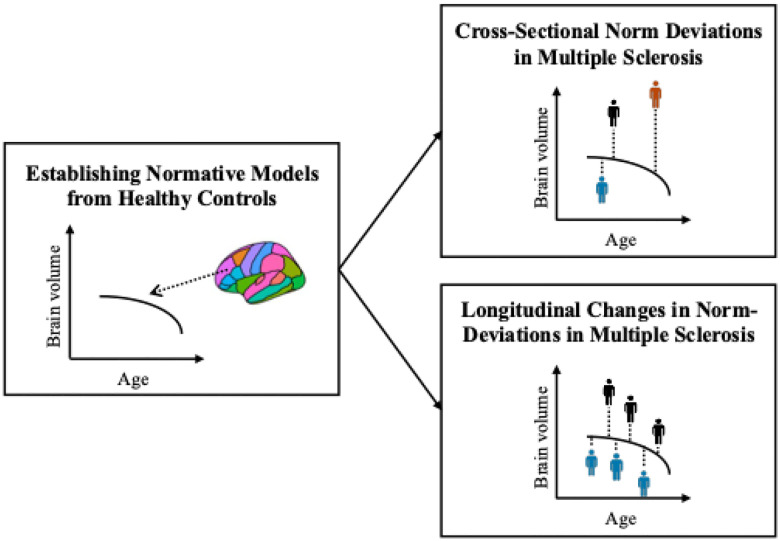
Analysis workflow. First, normative models were established on a large cohort of healthy controls covering the lifespan (6.0–90.1 years of age). Second, the reference models were applied to the MS cohort to assess norm-deviations cross-sectionally (upper right panel) and over time (lower right panel). The colour of the mannequins in the right panels represent a single individual with multiple sclerosis.

An overview of the full analysis workflow is presented in Figure 1.

#### 2.3.1 Model training

We used multivariate fractional polynomial regression,^24^ trained on regional brain volumes of the HC cohort. Models were trained for each sex separately, and trained for each brain region (i), where regional brain volumes were predicted from a linear effect of ICV, to account for confounding effects of head size, a fractional polynomial (p) of age (with maximum degree m=2), and an error term (u), with b_0-n_ indicating the regression coefficients.


$${\text{region}}_{i}=b_{0}+b_{1}{ICV}_{i}+b_{2}{age}^{p}+u_{i}$$

Model performance across regions was similar in training data R^2^ = 0.44±0.16, r = 0.66±0.13, root mean squared error (RMSE) (%) = 13.6±3.29 and mean absolute error (MAE) (%) = 10.6±2.58 compared to propensity matched HCs R^2^ = 0.34±0.19, r = 0.63±0.12, RMSE (%) = 14.80±3.96 and MAE (%) = 11.60±3.18. For an overview of region-wise model performance metrics see Supplemental Figure 7 and 8. Finally, to assess dataset-induced bias, we ran a leave-one-dataset-out analysis, where models were trained leaving out one of the utilised datasets (Supplemental Table 1) at a time. Across all regions, the Spearman correlations across all predictions were on average strong in training data (r = 0.99±0.02) and the propensity score matched HC (test) data (r = 0.99±0.03), used in case-control analyses.

#### 2.3.2 Associations between the number of deviations and clinical outcomes

The models were then used to predict the brain volumes per region in pwMS (N=362, T_1_w-scans=953), as well as in propensity-score matched cross-sectional HC sample (based on ICV, sex, and age, N=351). Z-scores, representing norm deviations, were calculated from the true volumes, predicted volumes, and model error (RMSE). First, we quantified the total number of regional significant deviations or extreme lower-than-reference values, defined by Z < −1.96. We then compared the number of XLTRVs per individual between pwMS and HC, using negative binomial regression to account for overdispersion. XLTRV was then associated with EDSS, PASAT and FSS scores at baseline, using cross-sectional linear models, and longitudinally, using linear random intercept models, controlling for disease duration (DD):

$$\text{Clinical}=b_{0}+b_{1}\text{XLTRV}+DD+u$$

Note that, by training subgroup specific models and including ICV in model-training, the Z-scores in test data are already sex, age, and ICV-adjusted; however, we corrected for DD. Moreover, we did not include information on treatment in the modelling as the treatment plans were not comparable to each other and highly variable (Supplemental Tables 4–5). This was due to the historic developments of treatments and treatment strategies. While there were 119 relapses, this number was restricted to 43 participants during the entire study period. Additionally, a large portion of these relapses were present during the first years of the OFAMS clinical trial (65 relapses across 37 patients) making this variable unsuitable as a covariate in longitudinal analyses. However, for quality control, we assessed the relationship between both Z-values and the number of extreme deviations with the number of relapses at baseline, presenting no significant relationships (Supplemental Note 3).

There was a small increase of T_1_-weighted lesions over the first two years of the study period. A two-year period was chosen to allow for comparability between samples. There were only 2 new T_1_-weighted lesions in Oslo data and 18 in OFAMS data. The count of T_2_-weighted lesions fluctuated over time. We did not observe associations between T_2_-weighted lesions and Z-values or extreme deviations, however significant associations were found for T_1_weighted lesions (Supplemental Note 3). Note however that T_1_-weighted lesions were not a significant predictor of EDSS and did not improve model performance compared to the number of extreme deviations (Supplemental Note 3). We did hence not include this variable in further analyses.

#### 2.3.3 Associations between the deviation magnitude (Z-scores) and clinical outcomes

Using the same logic from section 2.3.2, we assessed the impact of age, EDSS, PASAT and FSS on norm-deviations (Z) cross-sectionally and longitudinally.


$$\text{Clinical}=b_{0}+b_{1}Z_{\text{region}}+\text{DD}+u$$

Since extreme deviations were defined as |Z|>1.96, we defined stability of such deviations as the absence of any time transitions inside or outside the extreme deviation state (whether Z>1.96 or Z<−1.96).

#### 2.3.4 Deviation profile–based stratification

To evaluate the clinical utility of normative deviation profiles, we implemented a deviation-based stratification approach. A critical deviation was defined as any regional brain volume with a Z-score < –1.96 relative to the normative reference distribution. To derive an interpretable stratification rule grounded in the empirical deviation patterns observed in Section 3.2, we identified the three brain regions with the highest overlap of extreme deviations at baseline across pwMS, namely the bilateral thalamus, right superior parietal cortex, and pericalcarine cortex.

Participants were assigned to a deviation-based risk group if at least one critical deviation was present in any of these regions at baseline. For bilateral structures, a deviation in either hemisphere qualified. Risk status was treated as a baseline characteristic and held constant for all longitudinal analyses.

Clinical relevance of deviation-based risk was assessed using a) linear mixed-effects models for EDSS, PASAT, and FSS with random intercepts for subjects and adjustment for disease duration (and age and sex where appropriate), and b) recurrent-event survival models for clinical relapses in the OFAMS sub-sample. Relapse dynamics were modelled using Andersen-Gill Cox models with robust variance estimates clustered by subject, with sensitivity analyses performed using Prentice-Williams-Peterson gap-time models. Effect estimates are reported as regression coefficients or hazard ratios with 95% confidence intervals, alongside model concordance (C-index).

#### 2.3.5 Reporting and power

For comparability, we report standardized regression coefficients or effect sizes. The significance-level was set at a conventional alpha level=0.05, and the Benjamini-Hochberg (FDR) correction for multiple comparisons^25^ was applied for all tests. MRI data were used when also data on sex and age were available. This allowed sex-specific predictions and calculation of Z-scores using the normative models. Missingness was addressed using multiple imputation, when missingness did not exceed 50% of the data or was non-random. We used multivariate imputation by chained equations, using a single iteration of predictive mean matching across 5 imputed datasets. Due to missingness, least data were available for cross-sectional correlations between FSS scores and brain volumes. Here, the minimal observable effect was f^2^=0.25 (corresponding to R^2^=0.20, and Pearson’s r=0.45), at 80% power, alpha=0.05, 3 nominator and 43 denominator degrees of freedom. For statistical analyses, R version 4.5.2 was used. To represent spatial statistics for the examined brain areas, we used the ggseg R package.^26^

## 3. Results

### 3.1 Descriptives

A total of 362 people with MS (pwMS) contributed 953 T1-weighted MRI sessions acquired longitudinally over the study period. At baseline, 351 pwMS with available MRI data matched to healthy controls were aged 38.6±9.7 years, of whom 250 (71.2%) were female, with a mean disease duration of 4.66±6.15 years, mean EDSS of 2.0±1.2, PASAT score of 46.8±9.2, and mean FSS of 4.72±1.48.

Across the full dataset, 72 EDSS scores (7.76%) were missing and were imputed, resulting in complete clinical data for all 953 MRI sessions. A total of 119 relapses were recorded across 43 participants during follow-up. Additional baseline characteristics for sub-samples are reported in Supplementary Table 3, with Z-score distributions and extreme deviations shown in Supplementary Figures 1 and 11.

### 3.2 A temporally and spatially stable morphometric profile of multiple sclerosis

**Figure 2.**
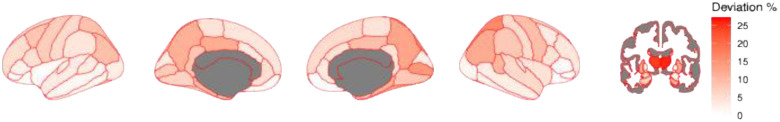
Overlap of significant deviations indicating lower brain volumes (Z<−1.96) across people with multiple sclerosis at baseline. Darker colours indicate a higher percentage of pwMS overlapping in lower-than-reference deviations in the respective brain region.

At baseline, the largest overlap of extreme lower-than-reference volumetric deviations across pwMS could be shown bilaterally in the thalami (25% and 26%, see Supplemental Figure 1 for distributions), followed by the superior parietal cortex (16%) and pericalcarine (14%; Figure 2). Multiple other regions presented extreme deviations in more than 10% of the pwMS, including the praecuneus, fusiform area, putamen, inferior parietal area, posterior cingulate, hippocampus and parahippocampal area. These regions also presented the strongest deviations, measured by Z-scores (Supplemental Figure 1). In contrast, brain volumes among matched HC corresponded to reference levels (Supplemental Figure 2).

Note that these deviations remained stable over time, indicated by maintaining the deviation status over time and across regions. On average, 94.81%±3.16% of the examined regions per participant did not show changes in the total number of extreme deviations, 2.96%±1.47% of the sample transitioned towards lower-than-reference brain volumes, and 2.39%±1.67% presented improvements, indicated by transitions from extreme lower-than-reference values towards non-extreme or extremely positive deviations. Note that less HC presented an accumulation of extreme lower-than-reference values (1.38±2.05%) than pwMS (Supplemental Note 2). Examples for individual-level profiles for Z-scores can be found in Supplemental Figure 9 and for Z-score-based extreme deviations in Supplemental Figure 10.

### 3.3 Associations between extreme lower-than-reference deviation count and clinical assessments

At baseline, extreme lower-than-reference brain volumes indicated higher EDSS (β=0.24, 95% Confidence Interval (CI) [0.14; 0.34], p<0.00001), but not PASAT (p=0.155), FSS (p=0.131), or disease duration (p=0.727). Similarly, longitudinally, the number of deviations increased with EDSS (β=0.07, 95% CI [0.02; 0.13], p = 0.016), but was not associated with PASAT (p=0.591), or FSS (p=0.255). Group-level cross-sectional case-control comparisons presented that pwMS had nearly three times the number of norm-deviations (4.51±4.95) compared to HC (1.67±2.67), indicated by an incidence rate ratio of 2.70, 95% CI [2.21; 3.30], p < 0.00001, and lower Z-scores than HC (average of regional mean absolute ΔZ=−0.22 (SE 0.48), d=−0.48, 95% CI [−0.17; −0.79], p=0.003). The regions with the largest negative Z-scores across pwMS, indicating smaller brain volumes, were the thalami (Z_left_=−1.16, Z_right_=−0.84), praecuneus (Z_left_=−0.94, Z_right_=−0.94), posterior cingulate (Z_left_=−0.91, Z_right_=−0.70), and putamen (Z_left_=−0.87, Z_right_=−0.16).

### 3.4 Associations between the magnitude of norm-deviations and clinical assessments

**Figure 3.**
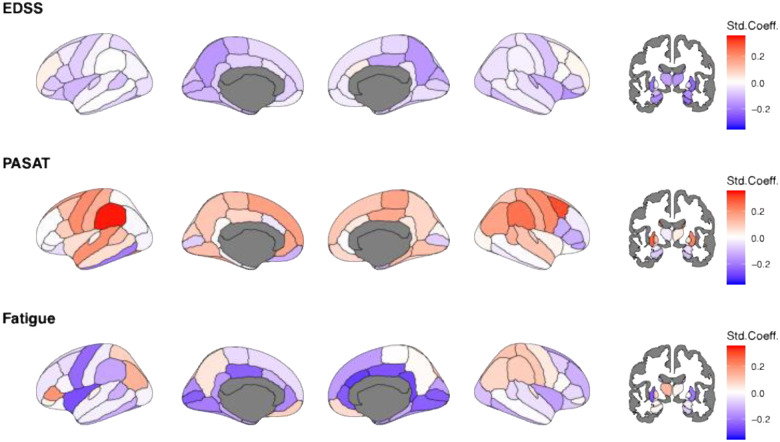
Baseline associations of age, EDSS, PASAT, and FSS on regional Z-scores. Std.Coeff.=standardized coefficient, EDSS= Expanded Disability Status Scale, PASAT= Paced Auditory Serial Addition Test, FSS=Fatigue Severity Scale. Note that only N=111 complete cases were available for PASAT and N = 119 for FSS scores. Considering the high level of missingness, imputation was not executed. Darker red colour indicates positive and darker blue colour negative norm-deviations, representing larger and smaller brain volumes compared to the reference. White colour indicates effects equal zero. Grey colour indicates uncorrected p>0.05.

At baseline, multiple regional deviations were significantly associated with EDSS and FSS, but not PASAT (p_FDR_>0.05; Figure 3). Significant associations were found between EDSS and volumetric deviations in cortical and subcortical regions, including the hippocampi (left: β_EDSS_=–0.17 95% CI [−0.27; −0.07]; p_FDR_=0.036; right: β_EDSS_=–0.21 95% CI [−0.31; −0.11], p_FDR_=0.004), right thalamus (right: β_EDSS_=−0.18 95% CI −0.29 to −0.07; p_FDR_=0.044), putamen (left: β_EDSS_=−0.21 95% CI [−0.31; −0.11], p_FDR_=0.004, right: β_EDSS_=−0.23 95% CI [−0.33, −0.07], p_FDR_=0.002), and right amygdala (β_EDSS_=−0.17 95% [−0.28; −0.07], p_FDR_=0.036).

Deviations from the cortical volumes in the left supramarginal gyrus (β_PASAT_=0.31 95% CI [0.13; 0.48], p_FDR_=0.010) predicted PASAT, and in the right isthmus cingulate (β_FSS_=−0.33 95% CI [−0.50; −0.16], p_FDR_=0.010) predicted FSS.

**Figure 4.**
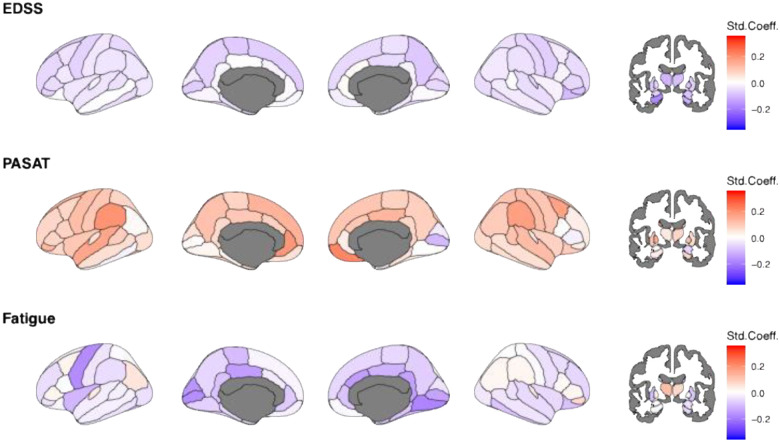
Longitudinal association of age, EDSS, PASAT, and FSS with regional Z-scores Std.Coeff.=standardized coefficient, EDSS= Expanded Disability Status Scale, PASAT= Paced Auditory Serial Addition Test, FSS=Fatigue Severity Scale. Age and EDSS were available for all participants, for N=107 (222 sessions) for PASAT and N=110 (214) for FSS, with this high level of missingness not allowing for imputation. Darker red colour indicates positive and darker blue colour negative norm-deviations, representing larger and smaller brain volumes compared to the reference. White colour indicates effects equal zero. Grey colour present regions which were not assessed. All associations were significant *before* FDR-corrections. Corrected, significant relationships are reported in-text.

In longitudinal analyses, lower Z-values, indicating lower-than-reference brain volumes, affected widespread and yet function-specific areas, with significant relationships detected for EDSS (Figure 4). EDSS could be predicted by lower-than-reference volumes in the thalami (left: β_EDSS_=−0.15 95% CI [−0.23; −0.08]; p_FDR_=0.006; right: β_EDSS_=−0.14 95% [CI −0.22; −0.07]; p_FDR_=0.021), hippocampi (left: β_EDSS_=−0.16 95% CI [−0.22; −0.10]; p_FDR_<0.001; right: β_EDSS_=−0.15 95% CI [−0.22; −0.07]; p_FDR_=0.021), and the left putamen (β_EDSS_=−0.18 95% CI [−0.26; −0.11]; p_FDR_=0.043). No reference values could significantly predict FSS or PASAT scores.

The results presented in sections 3.2–3.4 remained unchanged when applying a harmonisation strategy directly to the test data, except for counter-intuitive findings of lower levels of PASAT being associated with negative Z-scores (brain volumes smaller than the norm; see Supplemental Figures 3–6).

#### 3.4.5 Deviation profile-based stratification

Using the predefined deviation-based stratification rule, pwMS with at least one critical deviation in high-overlap regions exhibited higher disability over time compared to those without critical deviations.

##### 3.4.5.1 Group differences in EDSS, PASAT and FSS

Linear mixed models suggested higher EDSS over time in the deviation-based risk group (β_EDSS_ = 0.13 95% CI [0.03; 0.24], p = 0.01) when correcting for disease duration, but not PASAT (p = 0.43) or FSS (p = 0.41).

##### 3.4.5.2 Relapse risk and recurrence

To quantify the association between deviation-based risk group and relapse dynamics, we modelled time to relapse using survival analysis for recurrent events in the OFAMS sub-sample. In an Andersen-Gill Cox model with robust variance estimates clustered by subject, the presence of ≥1 critical deviation was associated with an increased relapse hazard (hazard ratio [HR] = 1.60, 95% CI [0.93; 2.74]; Wald p = 0.09). Results were highly consistent in a Prentice-Williams-Peterson gap-time model stratified by event order, yielding a comparable effect estimate (HR = 1.61, 95% CI [0.94; 2.76]; robust p = 0.08). Model concordance was modest in both frameworks (AG: C = 0.54; PWP: C = 0.54), reflecting limited discrimination at the individual level. Together, these results indicate a stable, directionally consistent association between critical deviations and higher relapse risk across recurrent-event modelling approaches, although effect estimates did not reach conventional levels of statistical significance.

## 4. Discussion

In this study, we applied large-scale normative models of regional brain volumes to longitudinal MRI data from pwMS and identified reproducible yet heterogeneous patterns of morphometric deviation relative to healthy population references. Extreme lower-than-reference brain volumes were unevenly distributed across regions, with the thalamus showing the largest and most consistent overlap across individuals. Approximately one quarter of the cohort exhibited extreme thalamic deviations, highlighting this structure as the single most representative region within the MS morphometric phenotype. At the same time, deviation profiles varied markedly between individuals, underscoring the heterogeneity of grey matter involvement in MS.

Both the magnitude of regional deviations, particularly in the thalamus, and the cumulative burden of extreme deviations were associated with disability cross-sectionally and longitudinally. Lower-than-reference volumes in subcortical structures, particularly the thalamus, hippocampus, and putamen, were consistently linked to higher disability, and in frontal and cingulate cortical regions to lower cognitive performance and higher fatigue. Although associations between thalamic atrophy and disability are well established in MS,^6–8^ expressing these abnormalities as age- and sex-adjusted normative Z-scores provides interpretable, individual-level metrics that extend beyond traditional group-level analyses.^1^ Beyond cross-sectional associations, deviations proved temporally stable. Extreme deviation status changed little over time, particularly in subcortical regions, suggesting that normative deviation profiles capture enduring disease-related traits rather than transient fluctuations. Nonetheless, transitions in deviation status, most notably in the thalamus, were observed in a subset of pwMS, emphasising the importance of repeated measurements for monitoring disease progression. Importantly, pwMS exhibited nearly three times the number of extreme deviations compared with matched healthy controls, and this ratio was stable over time, supporting the existence of a general MS-specific morphometric phenotype.

This phenotype, characterised by lower-than-reference-volumes in subcortical regions such as thalamus, putamen and limbic system, and occipital and parietal regions, such as superior parietal cortex, pericalcarine, praecuneus, and fusiform area, also provided a basis for clinically interpretable stratification. By defining deviation-based risk using regions with the largest overlap of extreme deviations, we identified patient subgroups with higher disability over time and a directionally consistent increase in relapse hazard. While relapse-related effects did not reach conventional significance thresholds, the consistency across recurrent-event models suggests that deviation profiles may capture aspects of disease vulnerability beyond relapse activity alone. These findings illustrate how normative modelling can translate high-dimensional imaging data into compact risk indicators suitable for clinical contexts.

Longitudinal analyses indicated that divergence from normative expectations increased with time, suggesting that normative models may help disentangle disease-related neurodegeneration from normative ageing processes.^3,27^ Biologically, thalamic involvement may reflect degeneration of long-range axonal connections given its role as a hub for major white matter tracts, whereas frontal cortical deviations may reflect processes such as accelerated ageing or sustained neuroinflammation.^28^

Normative modelling offers several advantages over conventional volumetric analyses. By enabling individual-level inference, it can reveal subclinical abnormalities not apparent on visual inspection or group averaging,^29^ making it particularly relevant for early disease stages and longitudinal monitoring. Integrating deviation profiles with established MRI markers such as lesion burden and global atrophy measures may improve patient stratification and support future precision-medicine approaches in MS.^29,30^ The broader applicability of this framework also suggests potential utility in other neurological and psychiatric disorders characterised by heterogeneous structural alterations.^31,32^

The application of normative models also opens avenues to better understand a) cases with diagnostic challenges, and b) the heterogeneity of disease trajectories in general. Similar to previously reported deviation patterns in psychiatric disorders,^31,32^ we found extensive heterogeneity across pwMS, yet with larger region-specific overlaps than in psychiatric disorders, especially in the thalamus. This underscores the potential of brain imaging assessments in MS and their further epidemiological investigation.

This study has several strengths, including the use of large, multi-site normative data, harmonised processing pipelines, robustness checks and independent longitudinal MS samples. As for limitations, normative models were limited to cross-sectional data, potentially limiting longitudinal inference. However, in contrast to other normative models such as BrainAge,^33–35^ the presented models could capture longitudinal processes related to disability progression. Additionally, brain-behaviour relationships are usually weak,^36^ which might be the reason for observed small and non-significant effects. Second, the test data presented in the main manuscript used for normative comparisons were not harmonised, which may introduce site-specific noise. On the other hand, this choice increases ecological validity and clinical applicability, and the results were stable also when computationally harmonising the test data prior to analyses. Third, clinical data (particularly PASAT and FSS) were sparsely available and missing at random, limiting the statistical power for some analyses, precluding imputation, and reducing our ability to include additional clinically meaningful covariates in the analyses. Moreover, treatments evolved over time introducing variability in the presented cohorts. Future studies with complete longitudinal assessments of cognitive and fatigue are needed to validate and extend these findings. Additionally, while we used validated segmentation pipelines, volume estimates may be affected by scanner and MRI protocol variability, particularly in longitudinal settings. Recent evidence suggests that lesion filling is superior to non-filling when estimating regional brain volumes and brain age, a type of normative modelling approach where age is the prediction target.^37^ However, since we found an association between T_1_-weighted lesions and both Z-values and the number of extreme deviations, we cannot differentiate whether the presence of lesions biases volumetric estimates in themselves influences the magnitude of the presented Z-scores, or whether there is a biologically meaningful associations. However, extreme deviations were stronger and significantly associated with EDSS, which was not the case for T_1_-weighted lesions, there was no significant interaction effect between EDSS and T_1_-weighted lesions, while multicollinearity was low (Supplemental Note 3). Still, our sensitivity analyses using harmonised data produced concordant trends, reinforcing the robustness of the primary findings (see Supplemental Note 4 for deviations). Future studies might consider additional segmentations of thalamic subfields or the medulla to further explore MS disability associated regions with population reference values.

In summary, by embedding regional brain volumes within a normative reference framework, we demonstrate that multiple sclerosis is characterised by a stable yet heterogeneous morphometric phenotype that is clinically meaningful at the individual level. Coordinated deviations, most prominently involving the thalamus and frontal cortical regions, relate to disability, cognitive function, and fatigue, and can be translated into compact deviation profiles that support patient stratification. While further validation in independent cohorts is required, these findings suggest that normative modelling provides a scalable and interpretable bridge between population-level neuroimaging and individual-level clinical assessment, with potential applications for early detection, monitoring, and personalised management of neurodegeneration in MS.

## Supplementary Material

Supplement 1

## Figures and Tables

**Figure 5.**
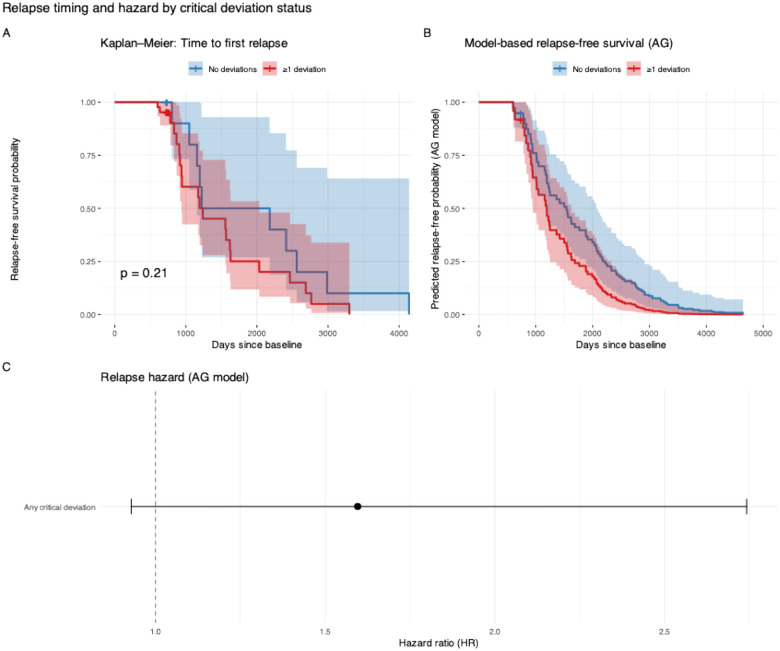
Relapse dynamics as a function of critical brain deviations. (A) Kaplan–Meier estimates of time to first clinical relapse, stratified by the presence of ≥1 critical deviation at baseline. Shaded areas indicate 95% confidence intervals. (B) Model-based relapse-free survival curves derived from an Andersen–Gill Cox model for recurrent events, comparing patients with and without critical deviations. (C) Forest plot summarising the hazard ratio for relapse associated with critical deviations from the Andersen–Gill model (points indicate hazard ratios; horizontal lines indicate 95% confidence intervals; dashed line denotes HR = 1). All Cox models used robust variance estimates clustered by subject to account for within-individual correlation.
