# Normative Modelling of Brain Volume in Multiple Sclerosis

**DOI:** 10.1101/2025.09.14.25335702

**Published:** 2026-04-07

**Authors:** Max Korbmacher, Ingrid Anne Lie, Kristin Wesnes, Eric Westman, Thomas Espeseth, Ole Andreas Andreassen, Lars T. Westlye, Stig Wergeland, Hanne Flinstad Harbo, Gro Owren Nygaard, Kjell-Morten Myhr, Einar August Høgestøl, Øivind Torkildsen, the Alzheimer’s Disease Neuroimaging Initiative

## Abstract

**Background and Objectives:**

Interpretation of brain atrophy in multiple sclerosis (MS) relies on group-level research and lacks individualized reference standards. Normative modelling can enable patient-level assessments of regional brain volumes relative to population expectations.

**Methods:**

We constructed age-, sex-, and intracranial volume–adjusted normative models of regional cortical and subcortical FreeSurfer-estimated brain volumes and applied to data from a concluded clinical trial and routine hospital examinations to derive regional deviation Z-scores and counts of critical deviations (Z < –1.96). Associations with disability (Expanded Disability Status Scale [EDSS]), cognitive performance (Paced Auditory Serial Addition Test [PASAT]), and fatigue (Fatigue Severity Scale [FSS]) were examined cross-sectionally and longitudinally using fixed and random effects models. A deviation-based stratification rule was evaluated for disability progression and relapse risk using survival analyses.

**Results:**

Models were trained on 62,444 MRI datasets from healthy individuals across the lifespan (50.8% females, age range 6.0-90.1) and applied to 953 longitudinal MRI scans from 362 people with MS (mean age = 38.8±9.7, 70.5% females, follow-up up to 12 years). People with MS exhibited a higher number of critical deviations than matched controls (incidence rate ratio 2.70, 95% CI 2.21–3.30), most prominently in the thalamus (approximately 25% of patients). A higher number of deviations was associated with higher disability (EDSS) indicated by a cross-sectional (β=0.24, 95% CI 0.14–0.34) and longitudinal main effect (β=0.07, 95% CI 0.02–0.13). Lower-than-reference volumes in the thalamus, hippocampus, and putamen were consistently associated with higher disability cross-sectionally (β _standardized_ = –0.17 to –0.23) and over time (β _standardized_ = –0.14 to –0.18). Deviation-based risk stratification identified patients with modestly higher disability trajectories (β=0.13, 95% CI 0.03–0.24).

**Discussion:**

Normative modelling reveals a heterogeneous morphometric deviation profile in MS, centred on deep grey matter structures and associated with disability accumulation. These findings support the use of population-referenced MRI metrics for individual-level phenotyping in MS and warrant validation in independent cohorts.

## Full Text Availability

The license terms selected by the author(s) for this preprint version do not permit archiving in PMC. The full text is available from the preprint server.

