# Supplementary material for: Normative Modelling of Brain Volume for Diagnostic and Prognostic Stratification in Multiple Sclerosis
Source: medRxiv. 2025 Nov 27:2025.09.14.25335702. Preprint. [Version 4] doi: 10.1101/2025.09.14.25335702 (PMC12458532; doi:10.1101/2025.09.14.25335702)
Supplement: Supplementary file 1 [file media-1.pdf]

Supplement

Article: Normative Models of Brain Volume for Diagnostic and Prognostic Stratification in Multiple Sclerosis, Korbmacher et al. (2025).

Contents

Supplemental Tables 2

Supplemental Table 1: Overview of the cross-sectional healthy control samples training data set 2

Supplemental Table 2: Overview of the OFAMS (multiple sclerosis) sample scanners and acquisition protocols 3

Supplemental Table 3: Baseline demographic and clinical characteristics of the pwMS cohorts. 5

Supplemental Table 4: Overview of administered treatments in Oslo data. 6

Supplemental Table 5: Overview of administered treatments in OFAMS (Bergen) data. 8

Supplemental Figures 11

Supplemental Figure 1. Distributions of the number of extreme deviations and Z-scores of norm deviations in the thalami in pwMS (non-harmonised test data) 11

Supplemental Figure 2. Region-averaged Z-values in MS and HC 12

Supplemental Figure 3. Overlap of significant deviations indicating lower brain volumes (Z<-1.96) across participants at baseline for harmonised data 13

Supplemental Figure 4. Region-averaged Z-values in MS and HC for harmonised data 14

Supplemental Figure 5: Overlap of significant deviations indicating lower brain volumes (Z<-1.96) across participants at baseline for harmonised data 15

Supplemental Figure 6. Longitudinal association of age, disability (EDSS), cognitive performance (PASAT), and fatigue on regional Z-scores for harmonised data 16

Supplemental Figure 7. Model performance: Variance explained and correlation coefficients for training (internal) and propensity matched (external) data. 17

Supplemental Figure 8. Model performance: Root mean squared error and mean absolute error for training (internal) and propensity matched (external) data. 18

Supplemental Figure 9. Individual-level Z-scores for minimum and maximum EDSS and disease duration 19

Supplemental Figure 9. Individual-level Z-score-based extreme deviations for minimum and maximum observed EDSS and disease duration 20

Supplemental Figure 11. Distribution of Z-scores at baseline in people with MS 21

Supplemental Figure 12. Individual-level Z-scores for minimum and maximum EDSS and disease duration using harmonised test data 22

Supplemental Figure 13. Individual-level Z-score-based extreme deviations for minimum and maximum observed EDSS and disease duration using harmonised test data 23

Supplemental Notes 24

Supplemental Note 1. Ethics Approvals 24

Supplemental Note 2. Longitudinal changes in training data with available longitudinal sessions. 25

Supplemental Note 3. Quality control analyses: associations between a) relapses, lesions, and b) Z-scores and extreme deviations. 26

Supplemental Note 4. Differences between main and sensitivity analyses. 28

References 29

Supplemental Tables

Supplemental Table 1: **Overview of the cross-sectional healthy control samples training data set**

**Data**	**Sample size**	**Mean age ± SD, range**	**FreeSurfer[1] version**
ABCD[2]	11 279	11.9±0.6, 10.6-13.6	5.3.0
AddNeuroMed[3]	292	74.2±6.2, 53.0-90.1	5.3.0
ADNI[4]	60	71.1±6.2, 56.6-86.4	7.4.1
Human Connectome Project[5] (HCP)[6]	1 097	28.8±3.7, 22.0-37.0	5.3.0
Rockland Sample[7]	827	35.3±20.9, 6.0-85.0	7.1.0
Thematically Organized Psychosis Research study [local Oslo data]	972	33.1±10.2, 13.0-72.0	7.4.1
UK Biobank[8]	48 040	64.9±7.8, 44.6-83.7	5.3.0

Healthy control training sample after removing missing data and not including the propensity-matched datasets: n=62 414. Superscript references can be found in the reference list at the end of the document.

Supplemental Table 2: **Overview of the OFAMS (multiple sclerosis) sample scanners and acquisition protocols**

**Protocol (N of patients)**	**1 (3)**	**2 (13)**	**3 (3)**	**4 (1)**	**5 (3)**	**6 (4)**	**7 (3)**	**8 (5)**
Scanner	Siemens Aera	Siemens Prisma	Siemens Skyra	Siemens Avanto	Siemens Skyra	Siemens Avanto	Siemens Aera	Philips Achieva
Field strength	1.5T	3T	3T	1.5T	3T	1.5T	1.5T	1.5T
Sequences	T1; MPRAGE T2; FLAIR	T1; MPRAGE T2; FLAIR	T1; MPRAGE T2; FLAIR	T1; MPRAGE T2; FLAIR	T1; MPRAGE T2; FLAIR	T1; MPRAGE T2; FLAIR	T1; MPRAGE T2; FLAIR	T1; FFE T2; FLAIR
TR (ms)	1940 5000	1800 5000	2300 5000	2060 5000	2300 5000	2200 6000	2200 5000	7.6 4800
TE (ms)	2.69 335	2.28 386	2.32 387	3.10 340	2.32 387	2.82 358	2.67 335	3.75 338
TI (ms)	976 1800	900 1800	900 1800	1100 1800	900 1800	900 2200	900 1800	1660 1650
Flip angle (¬∞)	8 120	8 120	8 120	15 120	8 120	8 120	8 120	8 90
Voxel size (mm)	1.00x0.98x0.98 1.00x1.00x1.00	1.00x1.00x1.00	1.00x1.00x1.00	1.00x1.00x1.00	0.9x0.94x0.94 0.9x0.45x0.45	1.00x0.49x0.49 1.00x0.51x0.51	0.90x0.94x0.94 0.90x0.45x0.45	1.00x0.98x0.98 1.00x1.00x1.00

**Protocol (N of patients)**	**9 (12)**	**10 (2)**	**11 (6)**	**12 (1)**	**13 (2)**	**14 (8)**	**15 (3)**
Scanner	Philips Achieva	Siemens Prisma	Philips Ingenia	Toshiba MRT200 SP3	Philips Ingenia	Siemens Prisma	Philips Achieva
Field strength	3T	3T	1.5T	1.5T	3T	3T	1.5T
Sequences	T1; FFE T2; FLAIR	T1; MPRAGE T2; FLAIR	T1; FFE T2; FLAIR	T1; FFE T2; FLAIR	T1; FFE T2; FLAIR	T1; MPRAGE T2; FLAIR	T1; FFE T2; FLAIR

	8.04	1800		13.5	11.11	1800	
TR (ms)	5000	5000	25 4800	1160	4800	5000	7.1 4800
TE (ms)	3.68 386	2.28 385	9.21 367	5.50 105	6.29 296	2.26 387	2.2 307
			1660				
TI (ms)	900 1800	900 1800	2300	1650 906	1800	1800	1660
Flip angle (¬∞)	8 90	8 120	30 90	20 90	8 90	8 120	8 90
							1.00x1.000x 1.00
							1.00x1.000x 1.00
	1.00x0.98x0.98	1.00x0.46x0.46	1.00x0.50x0.50	1.00x0.94x0.94	0.39x0.65x0.39	0.90x0.67x0.67	1.00x1.00x1.00
Voxel size (mm)	1.00x0.98x0.98	0.50x0.73x0.73	1.00x0.50x0.50	0.50x0.74x0.74	0.47x4.0x0.47	0.56x0.98x0.98	0.76x1.00x1.00

Supplemental Table 3: **Baseline demographic and clinical characteristics of the pwMS cohorts.**

	Total Sample (N=362)	OFAMS (N=84)	OUH (N=302)
Age	38.8 (9.7)	39.8 (8.4)	38.6 (9.9)
Female	256 (70.5%)	54 (64.3%)	220 (72.8%)
Male	107 (29.5%)	30 (35.7%)	82 (27.2%)
EDSS	2.0 (1.2)	1.9 (0.8)	2.0 (1.2)

Note that there was missingness at random for EDSS scores, and hence imputation was applied. The availability of other clinical assessments (PASAT and FSS) was limited at baseline, not allowing for imputation. MRI data were available for N=362 participants.

Supplemental Table 4: **Overview of administered treatments in Oslo data.**

**Patient**	**Session 1**	**Session 2**
	Unknown	Unknown
1	Teriflunomide	Alentuzumab
2	Fingolimod	Alentuzumab
3	None	None
4	None	Glatiramer Acetate
5	Fingolimod	Alentuzumab
6	Fumarate	Dimethylfumarate
7	Fingolimod	Fingolimod
8	Alentuzumab	Alentuzumab
9	Alentuzumab	Alentuzumab
10	Fingolimod	Fingolimod
11	Glatiramer Acetate	Glatiramer Acetate
12	None	None
13	Glatiramer Acetate	Glatiramer Acetate
14	None	None
15	Glatiramer Acetate	NA
16	Teriflunomide	Teriflunomide
17	Glatiramer Acetate	NA
18	Teriflunomide	Glatiramer Acetate
19	Fingolimod	Fingolimod
20	Teriflunomide	Teriflunomide
21	Fingolimod	Teriflunomide
22	Fingolimod	Fingolimod
23	None	None
24	Teriflunomide	Teriflunomide
25	None	Teriflunomide
26	None	None
27	None	None
28	Glatiramer Acetate	Glatiramer Acetate
29	None	None
30	Glatiramer Acetate	NA
31	Fingolimod	Fingolimod
32	None	None
33	Fingolimod	Fingolimod
34	Natalizumab	Natalizumab
35	Alemtuzumab	Alemtuzumab
36	None	Teriflunomide

37	Glatiramer Acetate	None
38	Glatiramer Acetate	Glatiramer Acetate
39	None	None
40	Teriflunomide	Teriflunomide
41	Glatiramer Acetate	NA
42	Teriflunomide	Teriflunomide
43	Interferon	Fingolimod
44	None	Teriflunomide
45	Fingolimod	Fingolimod
46	Alemtuzumab	Alemtuzumab
47	None	None
48	Teriflunomide	DimethylDimetylfumarate
49	None	NA
50	Fingolimod	Fingolimod
51	Dimetylfumarate	DimethylDimetylfumarate
52	Fingolimod	Fingolimod
53	Teriflunomide	Teriflunomide
54	Glatiramer Acetate	NA
55	Fingolimod	None
56	None	None
57	Teriflunomide	NA
58	None	NA
59	None	None

Supplemental Table 5: **Overview of administered treatments in OFAMS (Bergen) data.**

**Patient**	**DMT1 Name**	**DMT2 Name**	**DMT3 Name**	**DMT4 Name**	**DMT5 Name**
1	Rebif 44 mcg	Copaxone	Extavia		
2	Rebif 44 mcg				
3	Rebif 44 mcg				
4	Rebif 44 mcg				
5	Rebif 44 mcg	Copaxone			
6	Rebif 44 mcg	Copaxone			
7	Rebif 44 mcg	Rebif 44 mcg	Copaxone		
8					
9	Rebif 44 mcg				
10	Rebif 44 mcg	Copaxone			
11	Rebif 44 mcg	Tecfidera			
12	Rebif 44 mcg	Avonex	Copaxone		
13	Rebif 44 mcg				
14	Rebif 44 mcg				
15	Rebif 44 mcg	Rebif 22 mcg	Copaxone	Gilenya	
16	Rebif 44 mcg	Tysabri	Gilenya	Tysabri	
17	Tecfidera				
18	Rebif 44 mcg	Copaxone	Tysabri	HSCT Mexico	
19	Rebif 44 mcg	Copaxone	Tecfidera		
20	Rebif 44 mcg	Tysabri			
21	Rebif 44 mcg				
22	Rebif 44 mcg				
23	Copaxone				
24	Rebif 44 mcg				
25	Rebif 44 mcg	Gilenya			
26	Rebif 44 mcg	Copaxone 20 mg	Tysabri		
27	Rebif 44 mcg				
28	Rebif 44 mcg				
29					

30	Rebif 44 mcg				
31	Rebif 44 mcg				
32					
33	Avonex	Copaxone	Rebif 44 mcg	Tysabri	
34	Rebif 44 mcg	Rebif 22 mcg			
35					
36					
37	Rebif 44 mcg				
38	Rebif 44 mcg	Rebif 44 mcg	Aubagio		
39	Rebif 44 mcg	Copaxone	Tysabri		
40	Rebif 44 mcg	Aubagio			
41	Rebif 44 mcg	Aubagio			
42	Rebif 44 mcg	Rebif 44 mcg	Aubagio		
43	Rebif 44 mcg	Copaxone	Tysabri	Tecfidera	Gilenya
44					
45	Rebif 44 mcg				
46	Rebif 44 mcg	Copaxone			
47	Rebif 44 mcg	Copaxone			
48	Rebif 44 mcg	Avonex			
49	Rebif 44 mcg				
50	Rebif 44 mcg	Tecfidera			
51	Rebif 44 mcg	Copaxone	Gilenya		
52					
53	Rebif 44 mcg				
54	Rebif 44 mcg	Avonex	Tysabri	Gilenya	
55	Rebif 44 mcg				
56	Rebif 44 mcg	Copaxone 20,g	Tysabri	HSCT Moskva	
57	Rebif 44 mcg	Copaxone			
58	Rebif 44 mcg	Betaferon			
59	Rebif 44 mcg	Rebif 22 mcg			
60	Rebif 44 mcg				
61	Rebif 44 mcg	Mitoxantrone			

62	Rebif 44 mcg				
63	Rebif 44 mcg	Avonex	Tecfidera		
64	Rebif 44 mcg	Copaxone	Avonex		
65	Rebif 44 mcg	Tysabri			
66	Rebif 44 mcg	Tecfidera			
67	Rebif 44 mcg	Rebif 22 mcg	Copaxone	Tecfidera	
68	Rebif 44 mcg				
69	Rebif 44 mcg	Copaxone			
70	Rebif 44 mcg	Copaxone			
71	Rebif 44 mcg	Rebif 22 mcg	Copaxone		
72	Rebif 44 mcg	Copaxone 20 ,g	Aubagio		
73	Rebif 44 mcg	Rebif 22 mcg			
74	Rebif 44 mcg	IVIG	Gilenya	Tysabri	IVIG
75	Rebif 44 mcg	Tysabri			
76	Avonex	Tysabri	Gilenya		
77	Rebif 44 mcg	Tecfidera	Gilenya		
78	Rebif 44 mcg	Rebif 22 mcg	Copaxone		
79	Rebif 44 mcg	Rebif 22 mcg	Copaxone		
80	Rebif 44 mcg	Copaxone 20 mg			
81	Rebif 44 mcg	Copaxone			
82					
83	Rebif 44 mcg				
84	Rebif 44 mcg	Copaxone 20 mg	Copaxone 40 mg	Aubagio	
85	Rebif 44 mcg	Copaxone			

Supplemental Figures

Supplemental Figure 1. **Distributions of the number of extreme deviations and Z-scores of norm deviations in the thalami in pwMS (non-harmonised test data)**

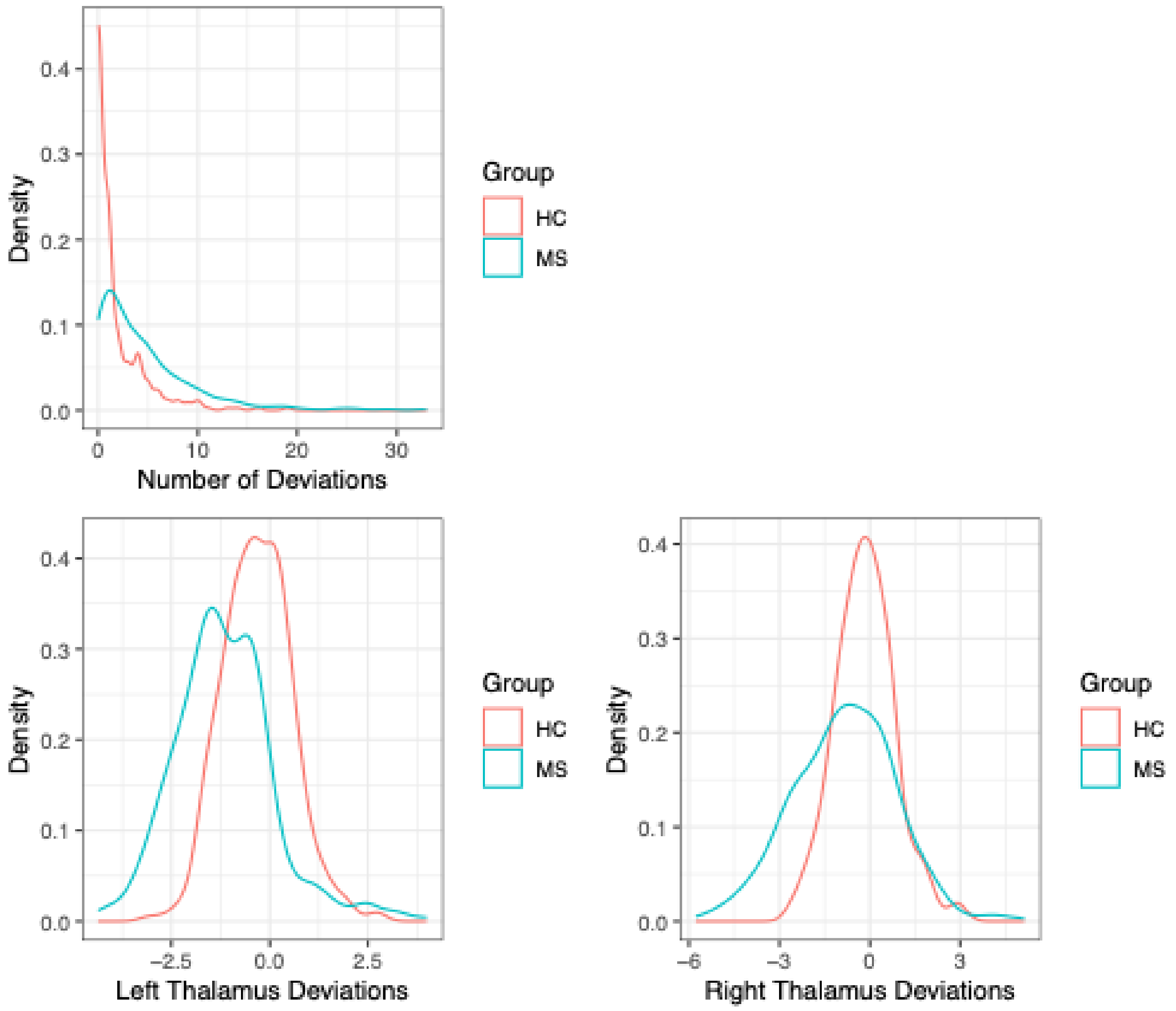

Top row: As reported in the main text, differences between the number of extreme deviations were significant between MS 4.51 (SE 4.95) HC 1.67 (SE 2.67), Cohen's d=0.71, 95% CI 0.56 to 0.87, p<0.0001. Bottom row: For the strongest effects, we show thalami Z-distributions. In pwMS, average deviations were Z_{left}=-1.16 and Z_{right}=-0.84, and for HC Z_{left}=-0.31 and Z_{right}=-0.15, being significantly different at Cohen's d_{left} = 0.77, 95% CI 0.62 to 0.93], p < 0.00001) and Cohen's d_{right} = 0.50, 95% CI 0.35 to 0.65], p < 0.00001).

Supplemental Figure 2. **Region-averaged Z-values in MS and HC**

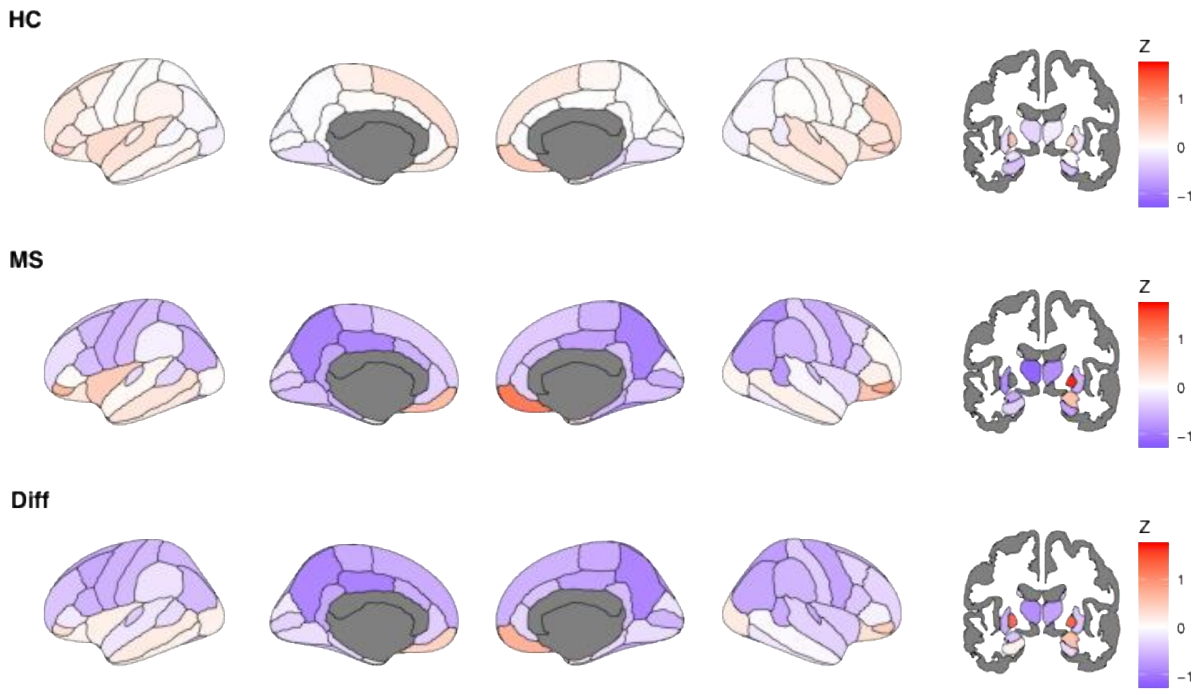

Regional Z-scores indicating deviations from the age-specific reference values in healthy controls (HC), multiple sclerosis (MS), and the delta between the examined samples. HC = healthy controls, MS = multiple sclerosis, Diff = difference between HC and MS.

Supplemental Figure 3. **Overlap of significant deviations indicating lower brain volumes (Z<-1.96) across participants at baseline for harmonised data**

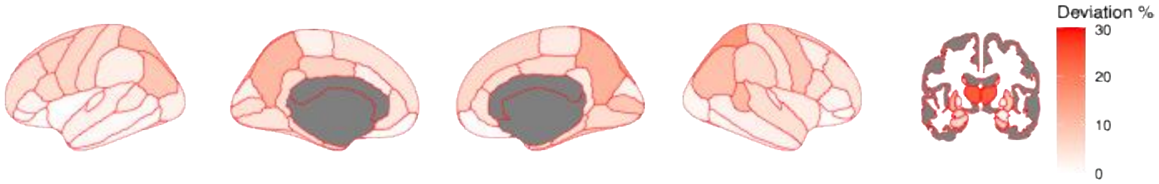

Supplemental Figure 4. **Region-averaged Z-values in MS and HC for harmonised data**

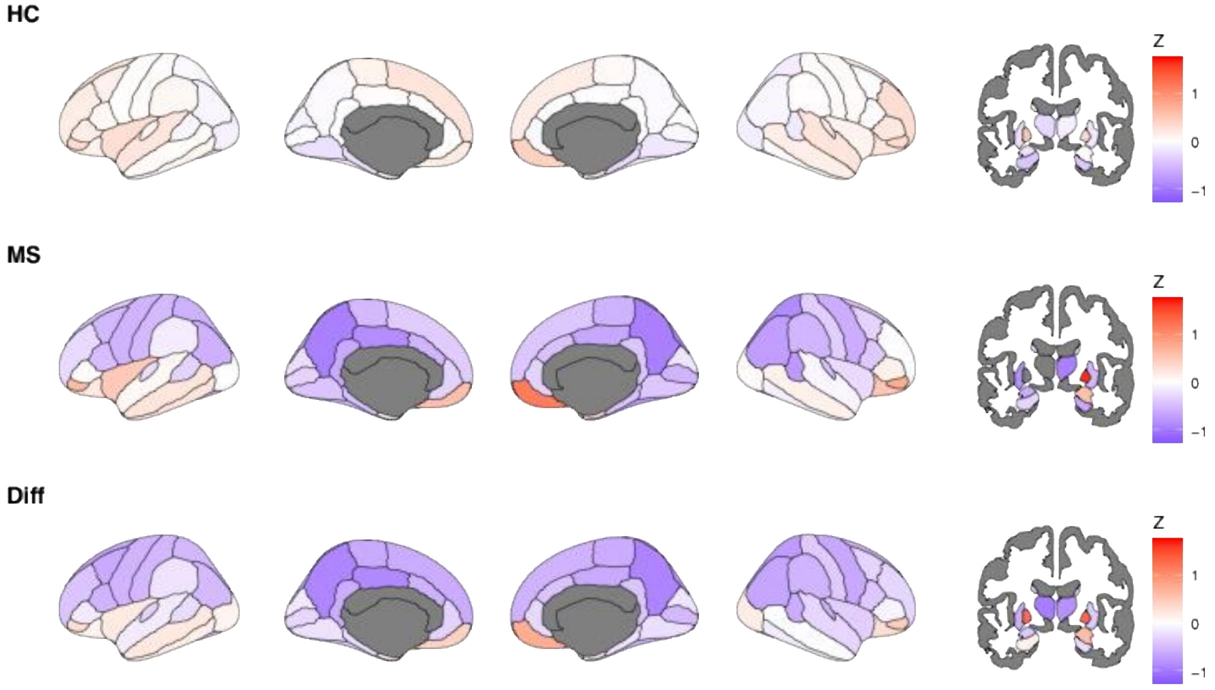

Regional Z-scores indicating deviations from the age-specific reference values in healthy controls (HC), multiple sclerosis (MS), and the delta between the examined samples. HC = healthy controls, MS = multiple sclerosis, Diff = difference between HC and MS.

Supplemental Figure 5: **Overlap of significant deviations indicating lower brain volumes (Z<-1.96) across participants at baseline for harmonised data**

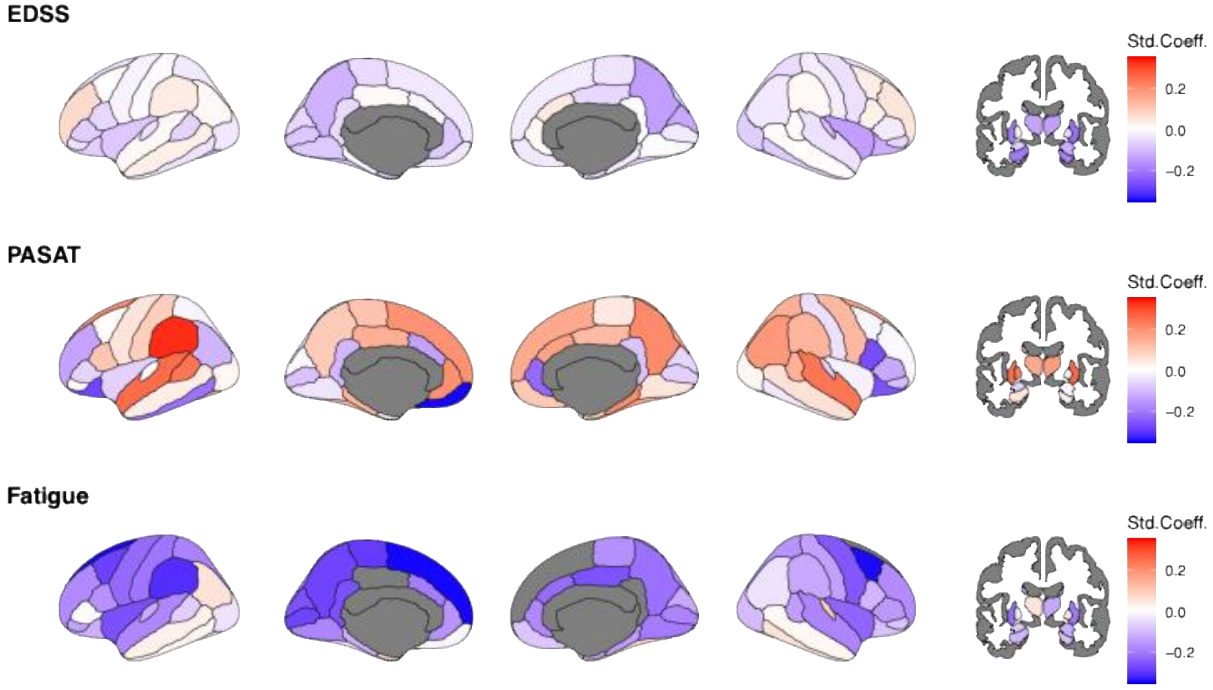

Std.Coeff. = standardized coefficient. Age and EDSS scores were available for N=214. Due to the harmonisation protocol leading to additional exclusions, tests were not possible for PASAT and FSS scores. The results generally correspond with the results observed in the main text, especially for associations of EDSS and subcortical volumes.

Supplemental Figure 6. **Longitudinal association of age, disability (EDSS), cognitive performance (PASAT), and fatigue on regional Z-scores for harmonised data**

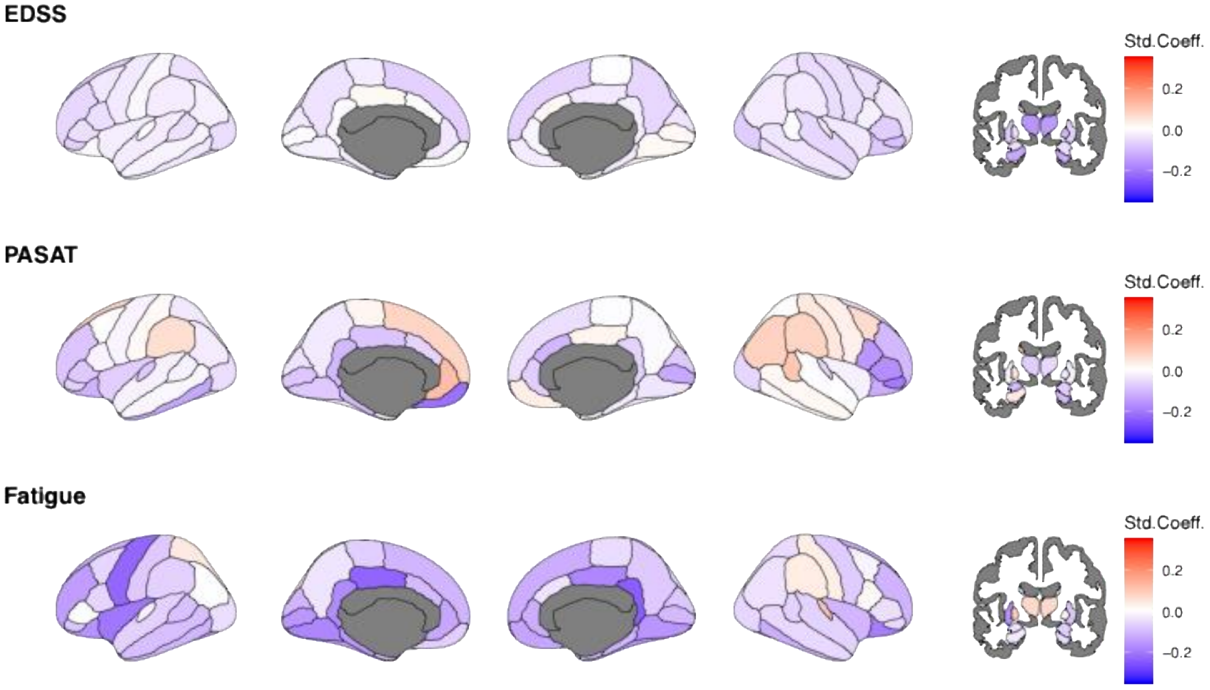

Std.Coeff. = standardized coefficient. Age and EDSS were available for 879 sessions (N=360). PASAT and fatigue scores were only available for N=95 (148 sessions) and N=133 (236 sessions), respectively. The high level of missingness did not allow imputations.

Supplemental Figure 7. **Model performance: Variance explained and correlation coefficients for training (internal) and propensity matched (external) data.**

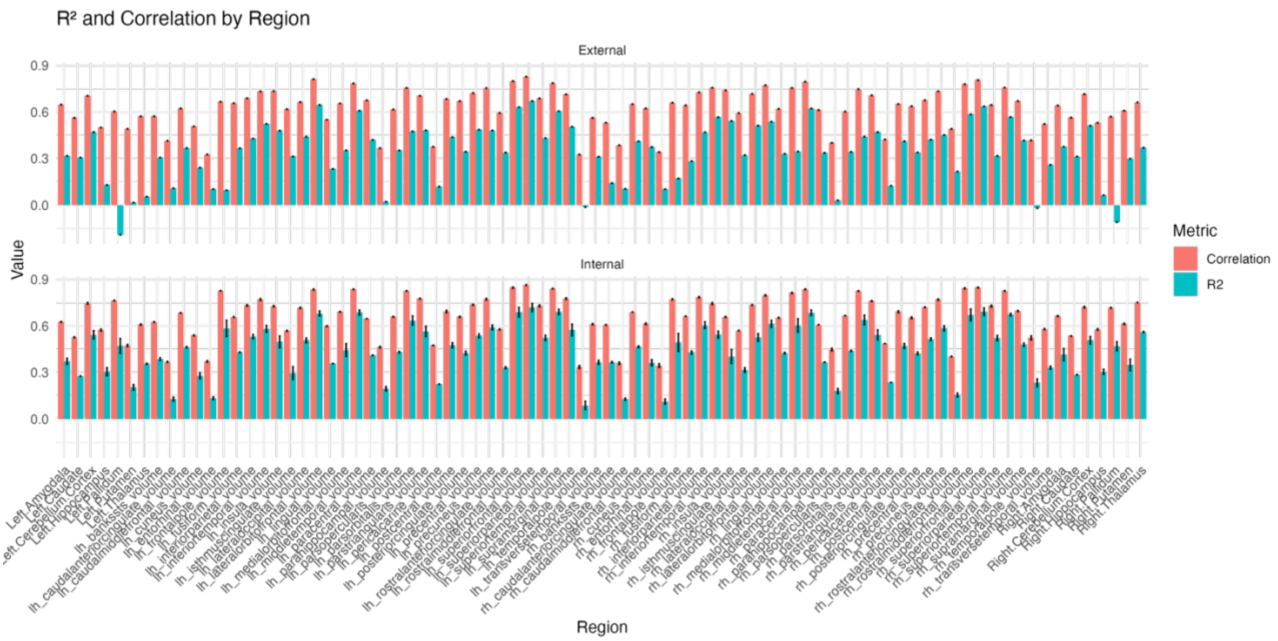

External data refer to the N = 351 propensity-matched healthy controls used for prediction. Internal data refers to the training data (N = 62 414).

Supplemental Figure 8. **Model performance: Root mean squared error and mean absolute error for training (internal) and propensity matched (external) data.**

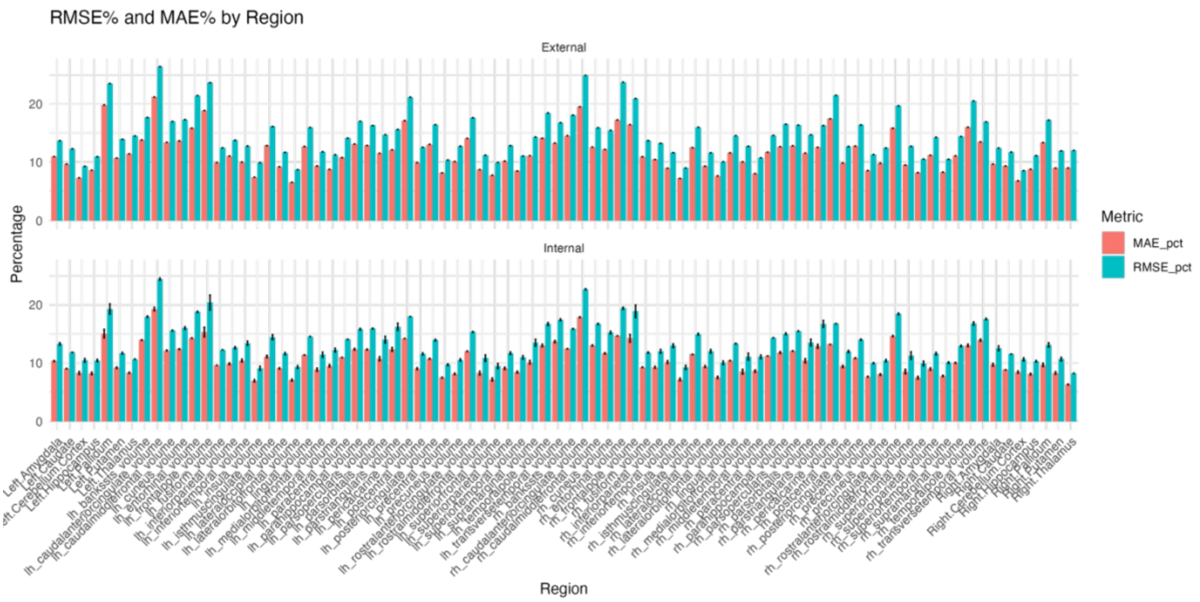

External data refer to the N = 351 propensity-matched healthy controls used for prediction. Internal data refers to the training data (N = 62 414).

Supplemental Figure 9. **Individual-level Z-scores for minimum and maximum EDSS and disease duration**

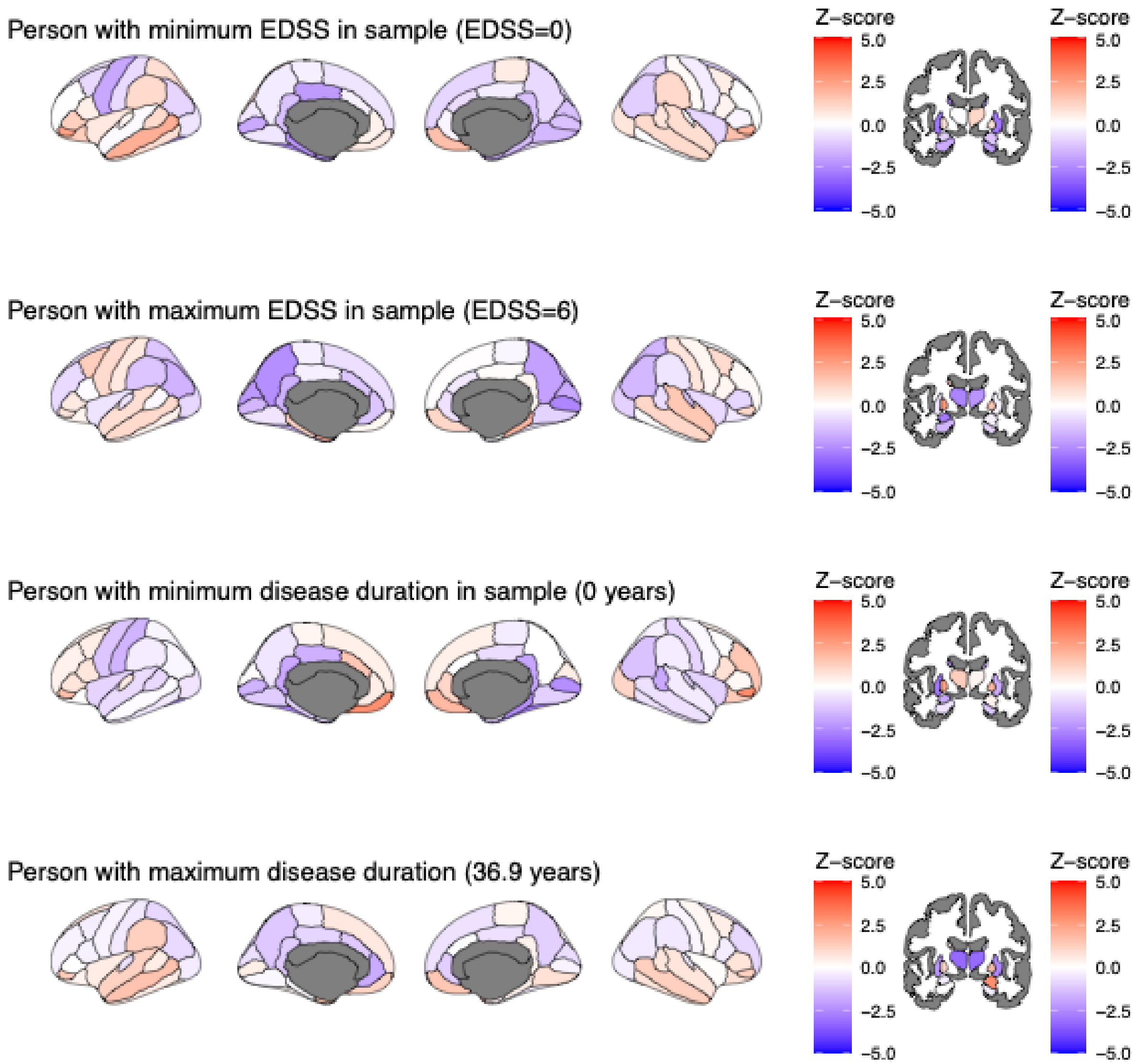

Supplemental Figure 9. **Individual-level Z-score-based extreme deviations for minimum and maximum observed EDSS and disease duration**

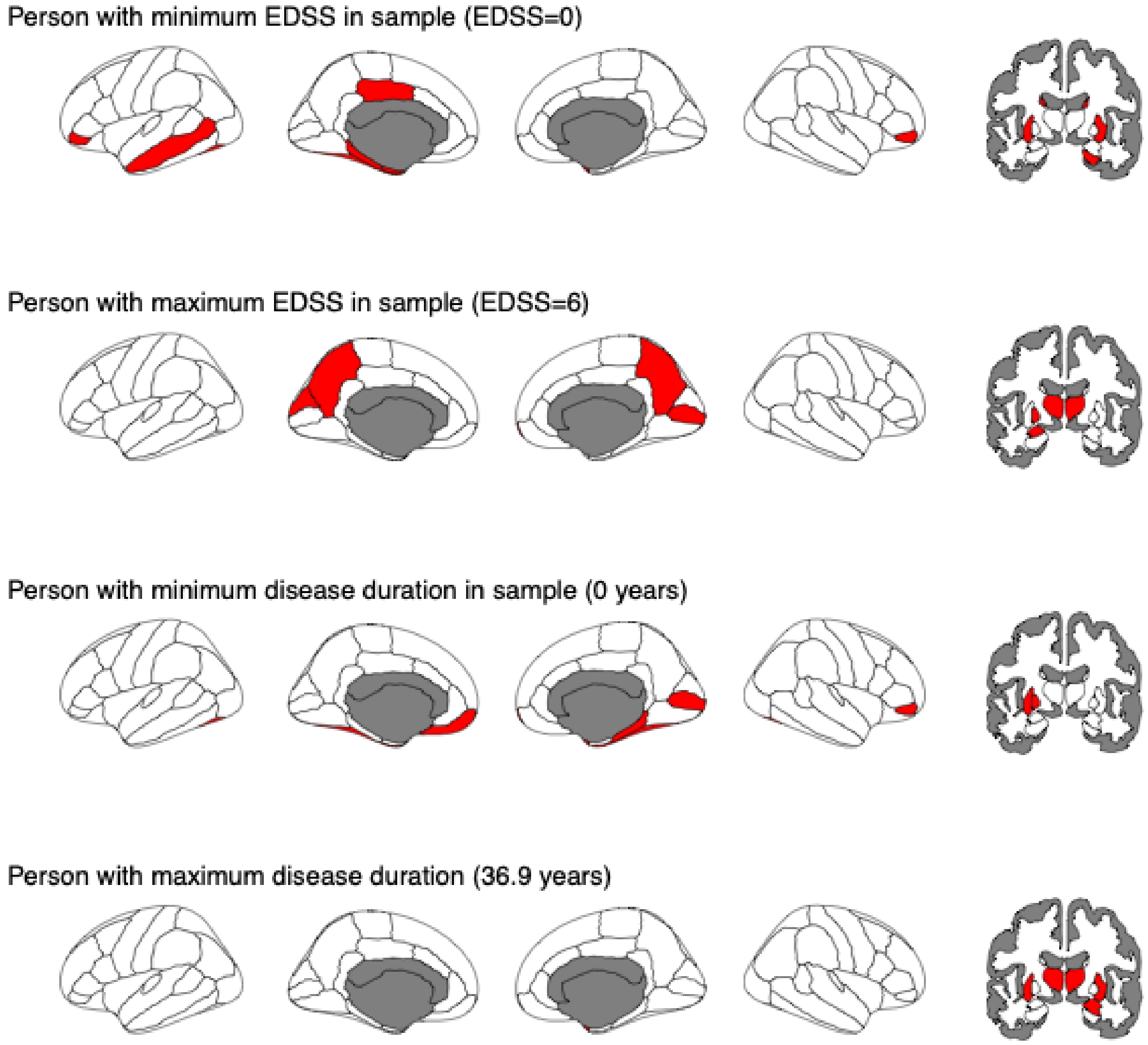

Extreme deviations were defined as Z ≤ -1.96.

Supplemental Figure 11. **Distribution of Z-scores at baseline in people with MS**

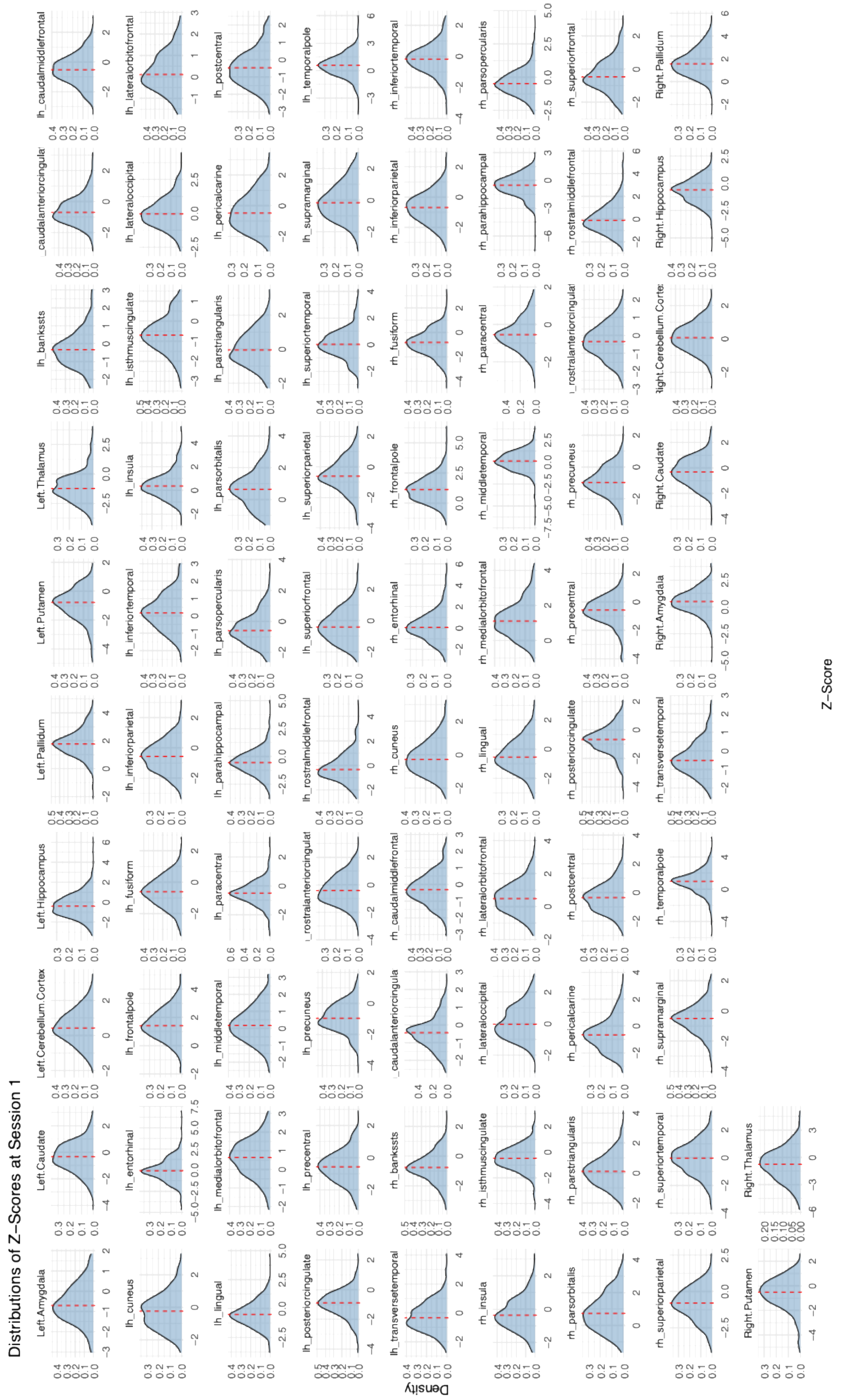

Supplemental Figure 12. **Individual-level Z-scores for minimum and maximum EDSS and disease duration *using harmonised test data***

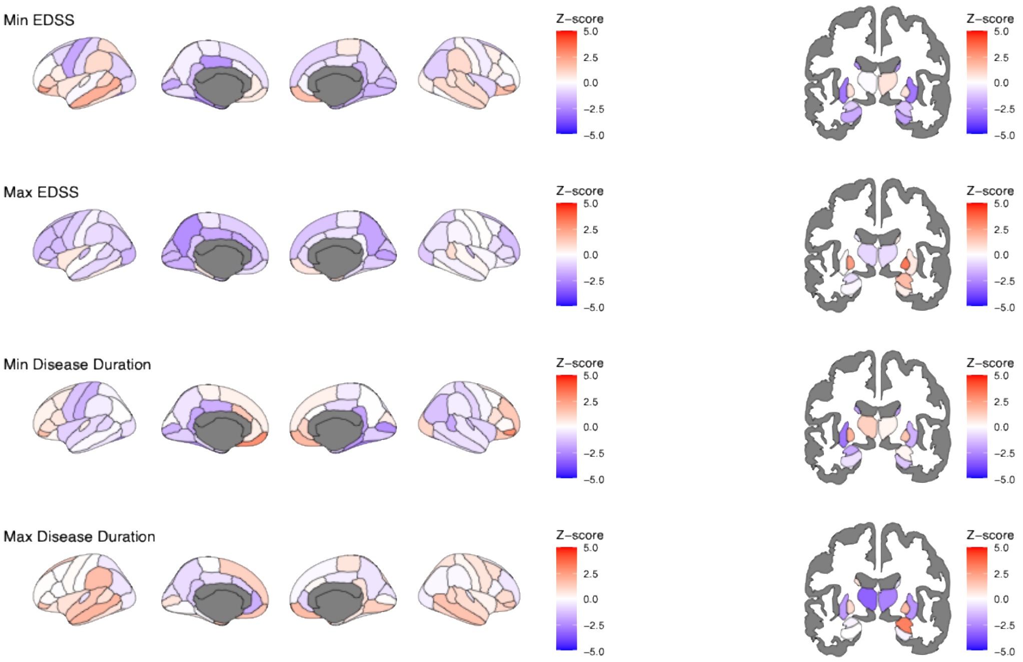

Supplemental Figure 13. **Individual-level Z-score-based extreme deviations for minimum and maximum observed EDSS and disease duration *using harmonised test data***

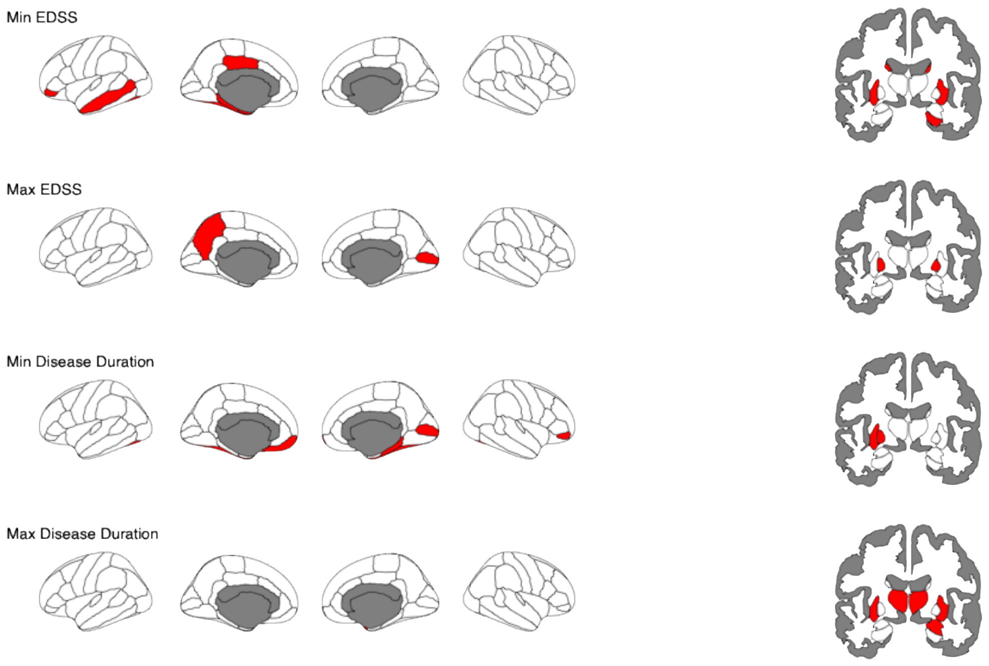

Supplemental Notes

Supplemental Note 1. **Ethics Approvals**

IRBs: **ABCD**: A centralised institutional review board approval of procedures was obtained from the University of California, San Diego. Written informed consent was obtained by a parent or guardian, and assent from the participants, before partaking in the ABCD study. **AddNeuroMed**: no IRB number available. **ADNI**: Data used in the preparation of this article were obtained from the Alzheimer's Disease Neuroimaging Initiative (ADNI) database (adni.loni.usc.edu). The ADNI was launched in 2003 as a public-private partnership, led by Principal Investigator Michael W. Weiner, MD. The primary goal of ADNI has been to test whether serial magnetic resonance imaging (MRI), positron emission tomography (PET), other biological markers, and clinical and neuropsychological assessment can be combined to measure the progression of mild cognitive impairment (MCI) and early Alzheimer's disease (AD). For up-to-date information, see www.adni-info.org. **HCP**: Ethics approval was obtained by the Norwegian Ethics Commission REK 567301, PVO 17/21624. Data used in the preparation of this work were obtained from the **Human Connectome Project (HCP)** database (https://ida.loni.usc.edu/login.jsp). The HCP project (Principal Investigators : Bruce Rosen, M.D., Ph.D., Martinos Center at Massachusetts General Hospital; Arthur W. Toga, Ph.D., University of Southern California, Van J. Weeden, MD, Martinos Center at Massachusetts General Hospital) is supported by the National Institute of Dental and Craniofacial Research (NIDCR), the National Institute of Mental Health (NIMH) and the National Institute of Neurological Disorders and Stroke (NINDS). HCP is the result of efforts of co-investigators from the University of Southern California, Martinos Center for Biomedical Imaging at Massachusetts General Hospital (MGH), Washington University, and the University of Minnesota. **Rockland Sample**: Ethics approval was obtained by the Norwegian Ethics Commission REK 567301, PVO 17/21624. **TOP**: The ethics approval was obtained by the Norwegian Ethics Commission REK 567301, PVO 17/21624. **UK Biobank**: Data were used under application 27412. Ethics approval was obtained from the National Health Service National Research Ethics Service (# 11/NW/0382).
An umbrella ethics approval (accross datasets) was obtained by the Norwegian Ethics Commission REK 567301, PVO 17/21624.

Supplemental Note 2. **Longitudinal changes in training data with available longitudinal sessions.**

To provide an understanding of how Z-values change over time in healthy controls, we estimated Z-scores in training data with longitudinal data available. These data included N = 2,395 participants from the (UK Biobank = 2,163) and the Rockland sample (N = 232), with a mean±SD follow-up duration of 2.26±0.86 years.

Across this time, healthy controls had an increase in extreme deviations of 1.33±1.68 across the 82 examined regions (1.38±2.05%), which was a lower number of changes than the examined changes in the MS cohort (2.96±1.47%). These changes were defined as a transition from not-lower-than extremely negative reference values ($Z > -1.96$) towards extremely negative deviations ($Z \leq -1.96$), which indicates accelerated atrophy beyond what would be expected from the normative models.

***Supplemental Note 3.* Quality control analyses: associations between a) relapses, lesions, and b) Z-scores and extreme deviations.**

For quality control, we assessed the a) relationships between relapses and both the Z-scores as well as the total number of extreme deviations, as well as b) the relationships between both b1) T1-weighted as well as b2) T2-weighted lesions and both the Z-scores as well as the total number of extreme deviations. These associations were controlled for disease duration, using linear fixed effects regression models with Z-scores or the total number of extreme deviations being the predictors. Finally, we used the same parameters to explain EDSS scores and evaluated model performance at the inclusion or exclusion of key parameters using Likelihood Ration Tests (LRT) and considered parameter estimates.

Associations of norm deviation, relapses and lesions

We did not observe a significant effect of the number of deviations on the number of relapses within 12 months prior baseline in the N = 118 participants with available data on the number of relapses (p = 0.564). We could neither observe an effect on the relapse rate over time (p = 0.474). None of the regional Z-scores were significantly associated with the number of relapses after FDA correction for multiple testing.

The number of T1-weighted lesions was significantly associated with the number of deviations (standardized coefficient = 0.28, 95% CI from 0.18 to 0.38, p < 0.000001) across participants (N = 364) indicating 0.09 added T1-weighted lesions with every added extreme deviation. Similarly, several regional Z-values were significantly associated with the number of T1-weighted lesions (see figure below).

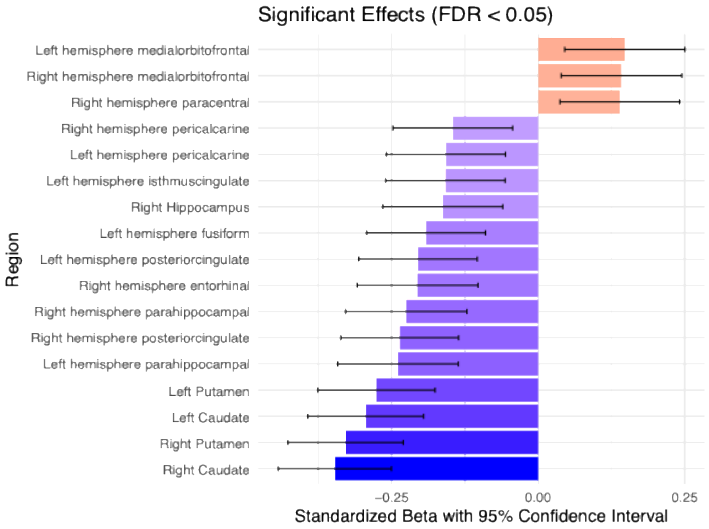

The number of T2-weighted lesions was however not associated with the number of extreme deviations at baseline (p=0.516). Yet, after FDA-correction region-level associations between T2-weigthted lesions and Z-scores in the right thalamus (standardized coefficient = -0.30, 95% CI from -0.47 to -0.12, p_{FDR} = 0.0469) and left superior frontal lobe (standardized coefficient = -0.31, 95% CI from -0.47 to -0.16, p_{FDR} = 0.0095) remained significant.

Predictors of EDSS

Finally, as a follow-up of the significant association between extreme deviations and T1-weighted lesions, we ran hierarchical regression models to examine the influence of either the number of T1-weighted lesions compared or the number of extreme deviations on EDSS. For that, we compared a full model predicting EDSS from T1-weighted lesions, the number of extreme deviations and the disease duration using LRT. First, T1-weighted lesions did not significantly predict EDSS, in any of the models (with ($p = 0.974$) or without ($p = 0.187$) including the number of extreme deviations in the model). Hence, removing T1-weighted lesions from the full model, did not significantly change model performance (LRT: $F = 0.001$, $p = 0.974$), and even indicated an increase in model performance, considering explained variance by $R^2 = 0.3\%$. However, removing the number of extreme deviations from the full model significantly lowered model performance ($F = 21.17$, $p = 0.000006$) from 9.09% of the variance explained to 3.95%. Standardized regression coefficients in the full model for the number of deviations were 0.24, 95% CI [0.14, 0.34], and for T1w lesions 0.0017, 95% CI [-0.10, 0.11]. Note that the variance inflation factor was below 1.1 across parameters across models, indicating the absence of multicollinearity.

For completeness, we also estimated models where EDSS was predicted by the number of extreme deviations, disease duration and either the number of T2-weighted lesions or the number of relapses at baseline. Both T2-weithted lesions ($p = 0.8696$ in the full model and $p = 0.9622$ in the model without number of extreme deviation) and the number of baseline relapses lesions ($p = 0.9557$ in the full model and $p = 0.944$ in the model without number of extreme deviation) were non-significant predictors of EDSS.

Supplemental Note 4. **Differences between main and sensitivity analyses.**
While slight variability in results from analyses considering harmonised compared to non-harmonised test data can be expected, we report here the findings where statistical tests would indicate different conclusions in sensitivity analyses compared to the analyses reported in the main text.

Magnitude of norm-deviations and clinical associations in cross-sectional analyses. Sensitivity analyses indicated that only hippocampi (left: β_{EDSS}=–0.20; p_{FDR}=0.014; right: β_{EDSS}=–0.19; p_{FDR}=0.021) and putamen (left: β_{EDSS}=–0.20, p_{FDR}=0.014, right: β_{EDSS}=–0.22, p_{FDR}=0.009) were significantly associated with EDSS, not thalami (p_{FDR}>0.05), in the cross-sectional modelling, where EDSS was predicted from region-level Z-scores considering disease duration as a fixed covariate. No regional deviation was significantly associated with processing speed (PASAT score; p_{FDR}>0.05), but superior frontal volume deviations were significantly associated with fatigue (FSS; β_{FSS} = -0.47; p_{FDR} = 0.014), when using the same model structure but predicting PASAT or FSS instead.

References

1. Fischl, B. FreeSurfer. *NeuroImage* **62**, 774–781 (2012).
2. Karcher, N. R. & Barch, D. M. The ABCD study: understanding the development of risk for mental and physical health outcomes. *Neuropsychopharmacology* **46**, 131–142 (2021).
3. Lovestone, S. *et al.* AddNeuroMed--the European collaboration for the discovery of novel biomarkers for Alzheimer's disease. *Ann. N. Y. Acad. Sci.* **1180**, 36–46 (2009).
4. Mueller, S. G. *et al.* Ways toward an early diagnosis in Alzheimer's disease: The Alzheimer's Disease Neuroimaging Initiative (ADNI). *Alzheimers Dement.* **1**, 55–66 (2005).
5. Elam, J. S. *et al.* The Human Connectome Project: A retrospective. *NeuroImage* **244**, 118543 (2021).
6. Van Essen, D. C. *et al.* The Human Connectome Project: A data acquisition perspective. *NeuroImage* **62**, 2222–2231 (2012).
7. Nooner, K. B. *et al.* The NKI-Rockland sample: a model for accelerating the pace of discovery science in psychiatry. *Front. Neurosci.* **6**, 152 (2012).
8. Alfaro-Almagro, F. *et al.* Image processing and Quality Control for the first 10,000 brain imaging datasets from UK Biobank. *NeuroImage* **166**, 400–424 (2018).
