# Supplementary material for: Normative Modelling of Brain Volume for Diagnostic and Prognostic Stratification in Multiple Sclerosis
Source: medRxiv. 2025 Nov 12:2025.09.14.25335702. Preprint. [Version 3] doi: 10.1101/2025.09.14.25335702 (PMC12458532; doi:10.1101/2025.09.14.25335702)
Supplement: Supplementary file 1 [file media-1.pdf]

Supplement

Article: Normative Models of Brain Volume for Diagnostic and Prognostic Stratification in Multiple Sclerosis, Korbmacher et al. (2025).

Supplemental Tables

Supplemental Table 1: **Overview of the cross-sectional healthy control samples training data set**

**Data**	**Sample size**	**Mean age ± SD, range**	**FreeSurfer[1] version**
ABCD[2]	11 279	11.9±0.6, 10.6-13.6	5.3.0
AddNeuroMed[3]	292	74.2±6.2, 53.0-90.1	5.3.0
ADNI[4]	60	71.1±6.2, 56.6-86.4	7.4.1
Human Connectome Project[5] (HCP)[6]	1 097	28.8±3.7, 22.0-37.0	5.3.0
Rockland Sample[7]	827	35.3±20.9, 6.0-85.0	7.1.0
Thematically Organized Psychosis Research study [local Oslo data]	972	33.1±10.2, 13.0-72.0	7.4.1
UK Biobank[8]	48 040	64.9±7.8, 44.6-83.7	5.3.0

Healthy control training sample after removing missing data and not including the propensity-matched datasets: n=62 414. Superscript references can be found in the reference list at the end of the document.

Supplemental Table 2: **Overview of the OFAMS (multiple sclerosis) sample scanners and acquisition protocols**

**Protocol (N of patients)**	**1 (3)**	**2 (13)**	**3 (3)**	**4 (1)**	**5 (3)**	**6 (4)**	**7 (3)**	**8 (5)**
Scanner	Siemens Aera	Siemens Prisma	Siemens Skyra	Siemens Avanto	Siemens Skyra	Siemens Avanto	Siemens Aera	Philips Achieva
Field strength	1.5T	3T	3T	1.5T	3T	1.5T	1.5T	1.5T
Sequences	T1; MPRAGE T2; FLAIR	T1; MPRAGE T2; FLAIR	T1; MPRAGE T2; FLAIR	T1; MPRAGE T2; FLAIR	T1; MPRAGE T2; FLAIR	T1; MPRAGE T2; FLAIR	T1; MPRAGE T2; FLAIR	T1; FFE T2; FLAIR
TR (ms)	1940 5000	1800 5000	2300 5000	2060 5000	2300 5000	2200 6000	2200 5000	7.6 4800
TE (ms)	2.69 335	2.28 386	2.32 387	3.10 340	2.32 387	2.82 358	2.67 335	3.75 338
TI (ms)	976 1800	900 1800	900 1800	1100 1800	900 1800	900 2200	900 1800	1660 1650
Flip angle (¬∞)	8 120	8 120	8 120	15 120	8 120	8 120	8 120	8 90
Voxel size (mm)	1.00x0.98x0.98 1.00x1.00x1.00	1.00x1.00x1.00	1.00x1.00x1.00	1.00x1.00x1.00	0.9x0.94x0.94 0.9x0.45x0.45	1.00x0.49x0.49 1.00x0.51x0.51	0.90x0.94x0.94 0.90x0.45x0.45	1.00x0.98x0.98 1.00x1.00x1.00

**Protocol (N of patients)**	**9 (12)**	**10 (2)**	**11 (6)**	**12 (1)**	**13 (2)**	**14 (8)**	**15 (3)**
Scanner	Philips Achieva	Siemens Prisma	Philips Ingenia	Toshiba MRT200 SP3	Philips Ingenia	Siemens Prisma	Philips Achieva
Field strength	3T	3T	1.5T	1.5T	3T	3T	1.5T
Sequences	T1; FFE T2; FLAIR	T1; MPRAGE T2; FLAIR	T1; FFE T2; FLAIR	T1; FFE T2; FLAIR	T1; FFE T2; FLAIR	T1; MPRAGE T2; FLAIR	T1; FFE T2; FLAIR

	8.04	1800		13.5	11.11	1800	
TR (ms)	5000	5000	25 4800	1160	4800	5000	7.1 4800
TE (ms)	3.68 386	2.28 385	9.21 367	5.50 105	6.29 296	2.26 387	2.2 307
			1660				
TI (ms)	900 1800	900 1800	2300	1650 906	1800	1800	1660
Flip angle (¬∞)	8 90	8 120	30 90	20 90	8 90	8 120	8 90
Voxel size (mm)	1.00x0.98x0.98<br>1.00x0.98x0.98	1.00x0.46x0.46<br>0.50x0.73x0.73	1.00x0.50x0.50<br>1.00x0.50x0.50	1.00x0.94x0.94<br>0.50x0.74x0.74	0.39x0.65x0.39<br>0.47x4.0x0.47	0.90x0.67x0.67<br>0.56x0.98x0.98	1.00x1.000x1.00<br>1.00x1.000x1.00<br>1.00x1.00x1.00<br>0.76x1.00x1.00

Supplemental Table 3: **Baseline demographic and clinical characteristics of the pwMS cohorts**.

	Total Sample (N=362)	OFAMS (N=84)	OUH (N=302)
Age	38.8 (9.7)	39.8 (8.4)	38.6 (9.9)
Female	256 (70.5%)	54 (64.3%)	220 (72.8%)
Male	107 (29.5%)	30 (35.7%)	82 (27.2%)
EDSS	2.0 (1.2)	1.9 (0.8)	2.0 (1.2)

Note that there was missingness at random for EDSS scores, and hence imputation was applied. The availability of other clinical assessments (PASAT and FSS) was limited at baseline, not allowing for imputation. MRI data were available for N=362 participants.

Supplemental Figures

***Supplemental Figure 1*. Distributions of the number of extreme deviations and Z-scores of norm deviations in the thalami in pwMS (non-harmonised test data)**

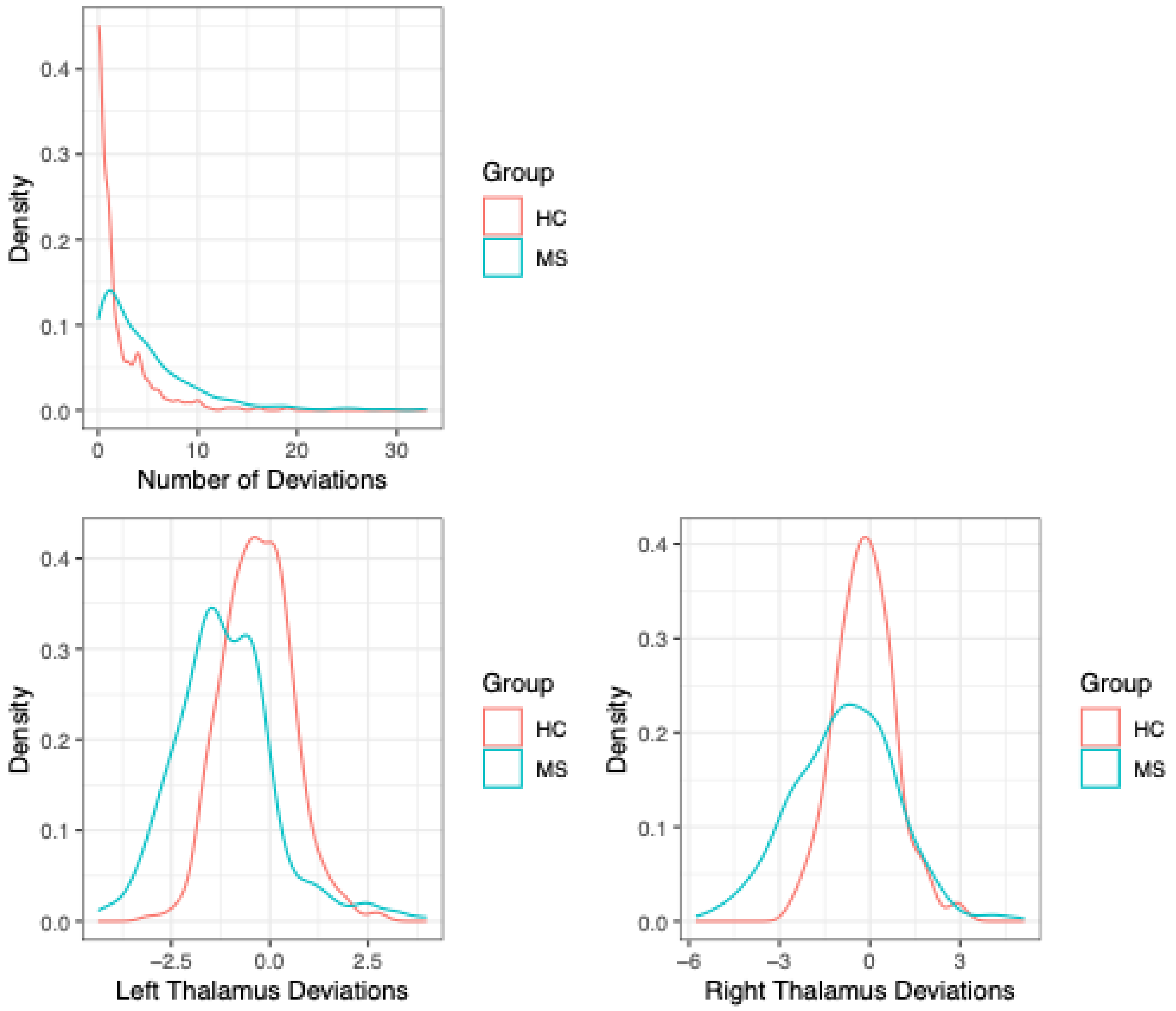

Top row: As reported in the main text, differences between the number of extreme deviations were significant between MS 4.51 (SE 4.95) HC 1.67 (SE 2.67), Cohen's d=0.71, 95% CI 0.56 to 0.87, p<0.0001. Bottom row: For the strongest effects, we show thalami Z-distributions. In pwMS, average deviations were Z_{left}=-1.16 and Z_{right}=-0.84, and for HC Z_{left}=-0.31 and Z_{right}=-0.15, being significantly different at Cohen's d_{left} = 0.77, 95% CI 0.62 to 0.93], p < 0.00001) and Cohen's d_{right} = 0.50, 95% CI 0.35 to 0.65], p < 0.00001).

***Supplemental Figure 2*. Region-averaged Z-values in MS and HC**

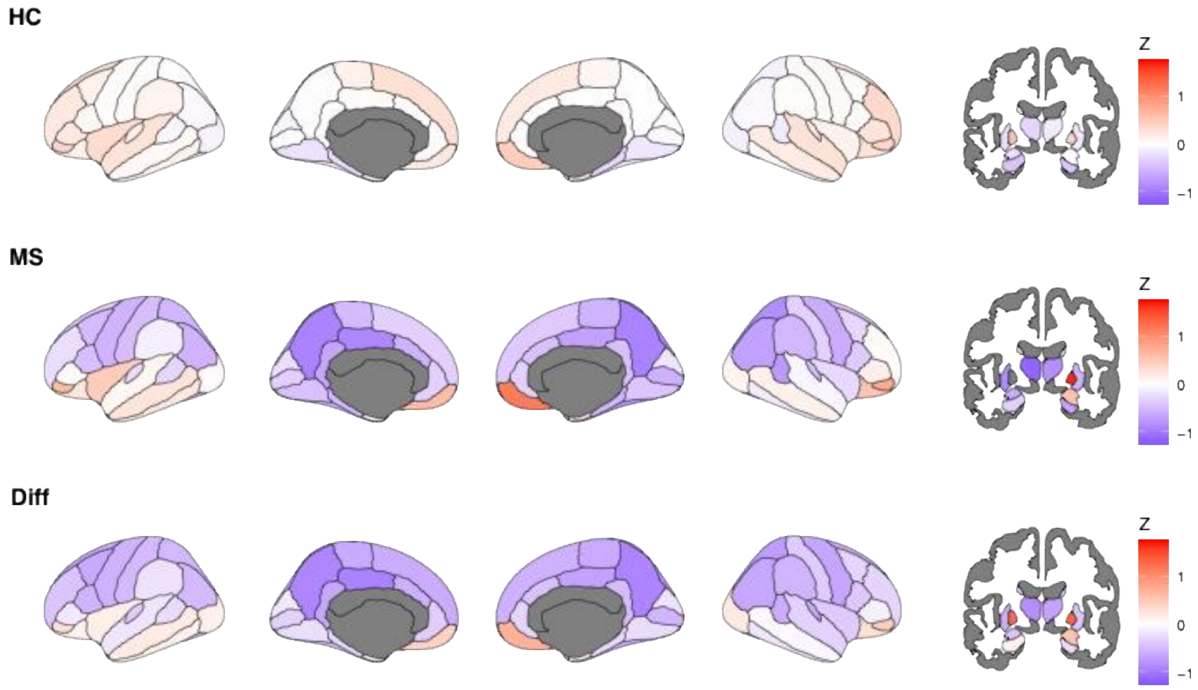

Regional Z-scores indicating deviations from the age-specific reference values in healthy controls (HC), multiple sclerosis (MS), and the delta between the examined samples. HC = healthy controls, MS = multiple sclerosis, Diff = difference between HC and MS.

***Supplemental Figure 3*. Overlap of significant deviations indicating lower brain volumes (Z<-1.96) across participants at baseline for harmonised data**

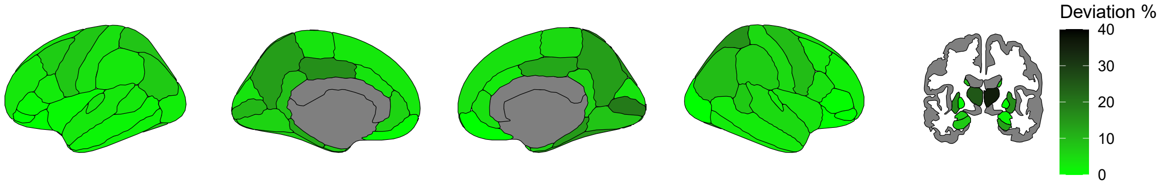

***Supplemental Figure 4*. Region-averaged Z-values in MS and HC for harmonised data**

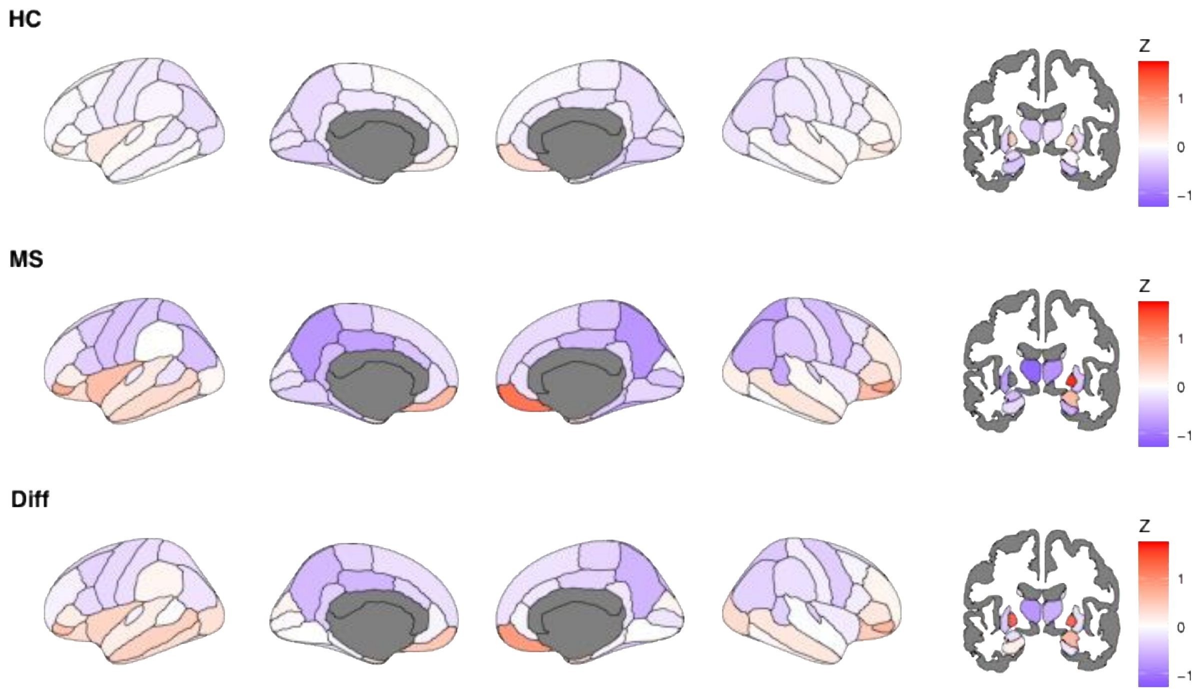

Regional Z-scores indicating deviations from the age-specific reference values in healthy controls (HC), multiple sclerosis (MS), and the delta between the examined samples. HC = healthy controls, MS = multiple sclerosis, Diff = difference between HC and MS.

Supplemental Figure 5: **Overlap of significant deviations indicating lower brain volumes (Z<-1.96) across participants at baseline for harmonised data**

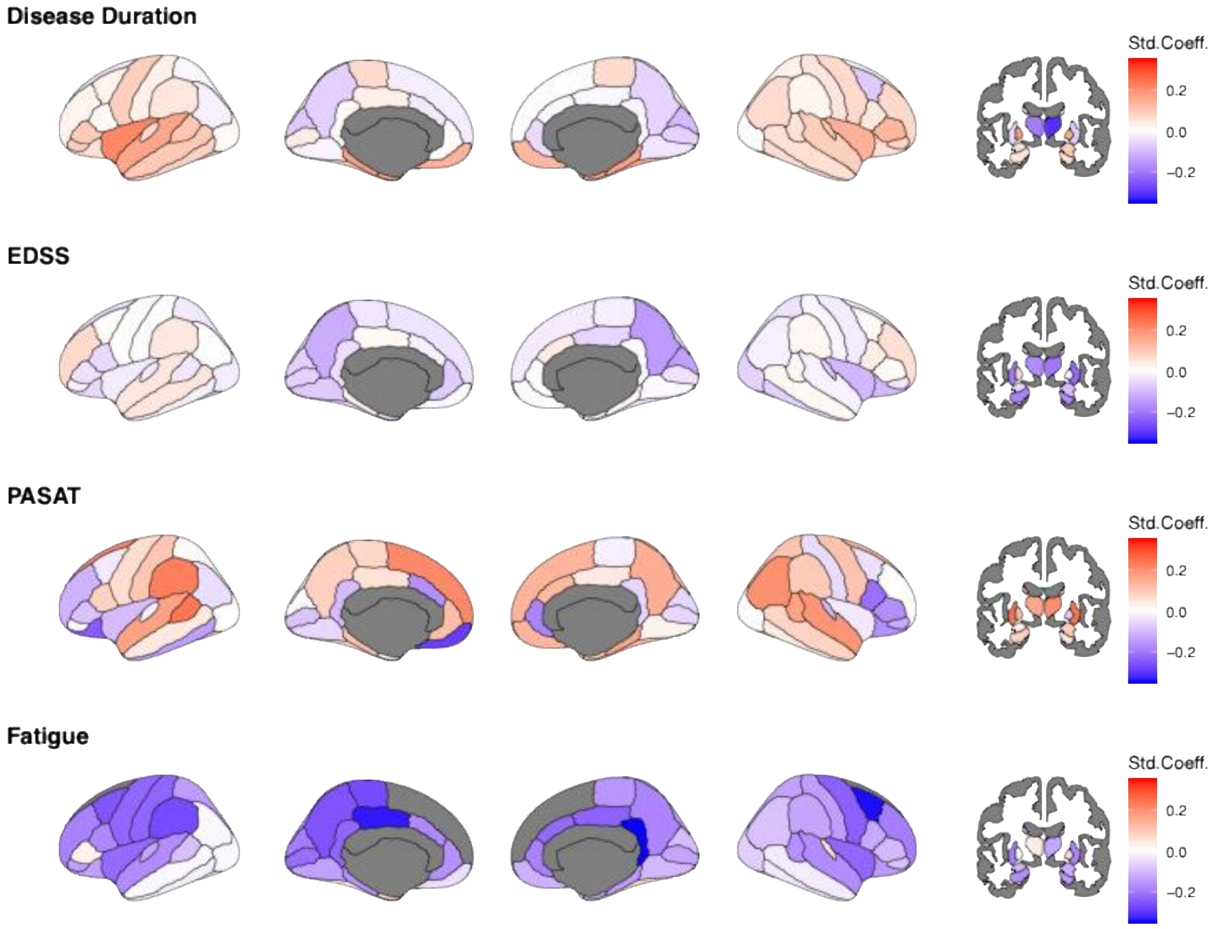

Std.Coeff. = standardized coefficient. Age and EDSS scores were available for N=214. Due to the harmonisation protocol leading to additional exclusions, tests were not possible for PASAT and FSS scores. The results generally correspond with the results observed in the main text, especially for associations of EDSS and subcortical volumes.

Supplemental Figure 6. **Longitudinal association of age, disability (EDSS), cognitive performance (PASAT), and fatigue on regional Z-scores for harmonised data**

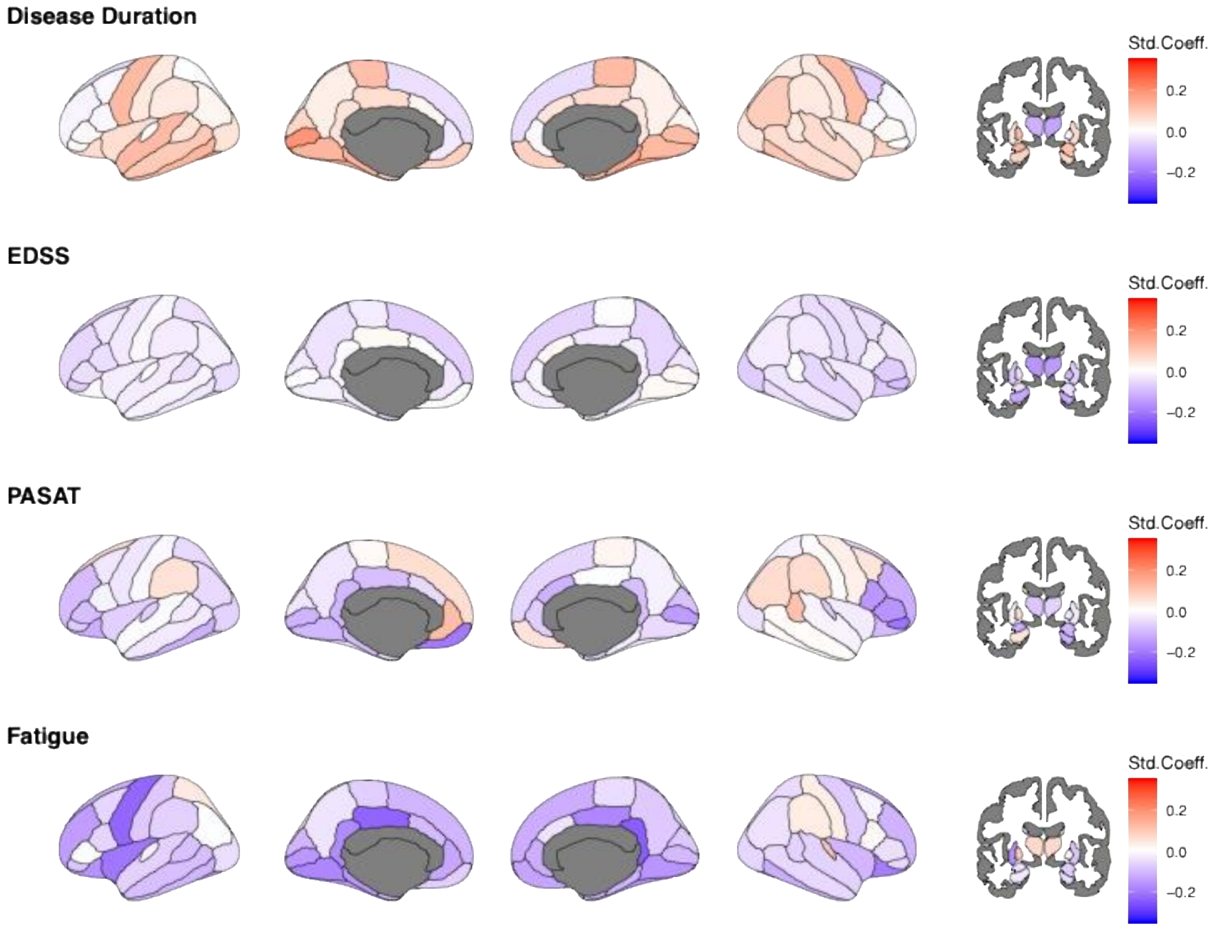

Std.Coeff. = standardized coefficient. Age and EDSS were available for 879 sessions (N=360). PASAT and fatigue scores were only available for N=95 (148 sessions) and N=133 (236 sessions), respectively. The high level of missingness did not allow imputations.

***Supplemental Figure 7*. Model performance: Variance explained and correlation coefficients for training (internal) and propensity matched (external) data.**

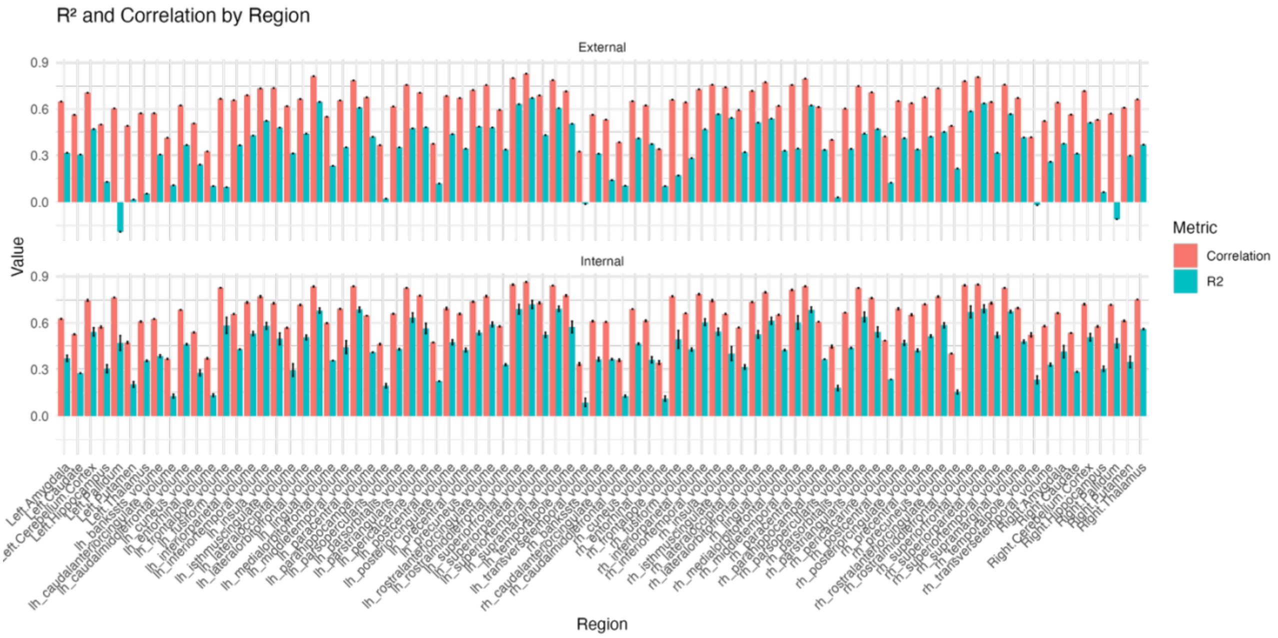

External data refer to the N = 351 propensity-matched healthy controls used for prediction. Internal data refers to the training data (N = 62 414).

***Supplemental Figure 8*. Model performance: Root mean squared error and mean absolute error for training (internal) and propensity matched (external) data.**

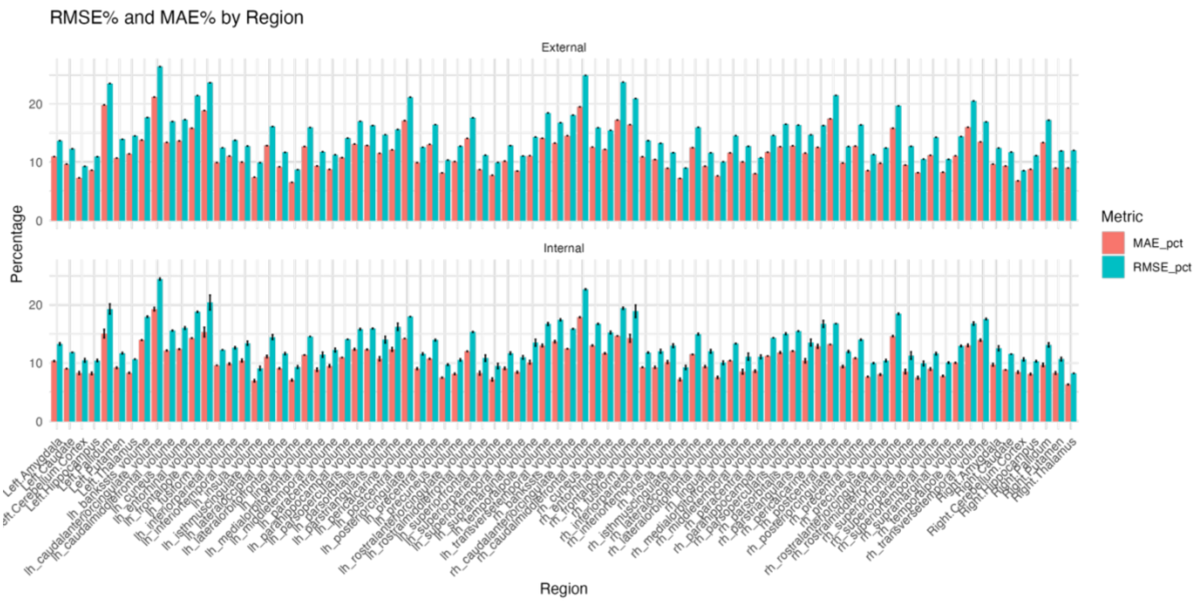

External data refer to the N = 351 propensity-matched healthy controls used for prediction. Internal data refers to the training data (N = 62 414).

Supplemental Note 1. **Ethics Approvals**

IRBs: **ABCD**: A centralised institutional review board approval of procedures was obtained from the University of California, San Diego. Written informed consent was obtained by a parent or guardian, and assent from the participants, before partaking in the ABCD study. **AddNeuroMed**: no IRB number available. **ADNI**: Data used in the preparation of this article were obtained from the Alzheimer's Disease Neuroimaging Initiative (ADNI) database (adni.loni.usc.edu). The ADNI was launched in 2003 as a public-private partnership, led by Principal Investigator Michael W. Weiner, MD. The primary goal of ADNI has been to test whether serial magnetic resonance imaging (MRI), positron emission tomography (PET), other biological markers, and clinical and neuropsychological assessment can be combined to measure the progression of mild cognitive impairment (MCI) and early Alzheimer's disease (AD). For up-to-date information, see www.adni-info.org. **HCP**: Ethics approval was obtained by the Norwegian Ethics Commission REK 567301, PVO 17/21624. Data used in the preparation of this work were obtained from the **Human Connectome Project (HCP)** database (https://ida.loni.usc.edu/login.jsp). The HCP project (Principal Investigators : Bruce Rosen, M.D., Ph.D., Martinos Center at Massachusetts General Hospital; Arthur W. Toga, Ph.D., University of Southern California, Van J. Weeden, MD, Martinos Center at Massachusetts General Hospital) is supported by the National Institute of Dental and Craniofacial Research (NIDCR), the National Institute of Mental Health (NIMH) and the National Institute of Neurological Disorders and Stroke (NINDS). HCP is the result of efforts of co-investigators from the University of Southern California, Martinos Center for Biomedical Imaging at Massachusetts General Hospital (MGH), Washington University, and the University of Minnesota. **Rockland Sample**: Ethics approval was obtained by the Norwegian Ethics Commission REK 567301, PVO 17/21624. **TOP**: The ethics approval was obtained by the Norwegian Ethics Commission REK 567301, PVO 17/21624. **UK Biobank**: Data were used under application 27412. Ethics approval was obtained from the National Health Service National Research Ethics Service (# 11/NW/0382).
An umbrella ethics approval (accross datasets) was obtained by the Norwegian Ethics Commission REK 567301, PVO 17/21624.

References

1. Fischl, B. FreeSurfer. *NeuroImage* **62**, 774–781 (2012).
2. Karcher, N. R. & Barch, D. M. The ABCD study: understanding the development of risk for mental and physical health outcomes. *Neuropsychopharmacology* **46**, 131–142 (2021).
3. Lovestone, S. *et al.* AddNeuroMed--the European collaboration for the discovery of novel biomarkers for Alzheimer's disease. *Ann. N. Y. Acad. Sci.* **1180**, 36–46 (2009).
4. Mueller, S. G. *et al.* Ways toward an early diagnosis in Alzheimer's disease: The Alzheimer's Disease Neuroimaging Initiative (ADNI). *Alzheimers Dement.* **1**, 55–66 (2005).
5. Elam, J. S. *et al.* The Human Connectome Project: A retrospective. *NeuroImage* **244**, 118543 (2021).
6. Van Essen, D. C. *et al.* The Human Connectome Project: A data acquisition perspective. *NeuroImage* **62**, 2222–2231 (2012).
7. Nooner, K. B. *et al.* The NKI-Rockland sample: a model for accelerating the pace of discovery science in psychiatry. *Front. Neurosci.* **6**, 152 (2012).
8. Alfaro-Almagro, F. *et al.* Image processing and Quality Control for the first 10,000 brain imaging datasets from UK Biobank. *NeuroImage* **166**, 400–424 (2018).
